# Ecological and socio-cultural factors influencing plant management in Náhuatl communities of the Tehuacán Valley, Mexico

**DOI:** 10.1186/1746-4269-9-39

**Published:** 2013-06-02

**Authors:** José Blancas, Alejandro Casas, Diego Pérez-Salicrup, Javier Caballero, Ernesto Vega

**Affiliations:** 1 Centro de Investigaciones en Ecosistemas (CIECO), Universidad Nacional Autónoma de México (campus Morelia), Antigua Carretera a Pátzcuaro 8711 Col. Ex Hacienda de San José de la Huerta, Morelia, Michoacán 58190, México; 2 Jardín Botánico, Instituto de Biología, Universidad Nacional Autónoma de México, Apdo. Postal 70-614, C.P. 04510, Ciudad Universitaria, D.F, México

**Keywords:** Domestication, Food Security, Plant Management, Risk Management, Tehuacán Valley

## Abstract

**Background:**

Management types and their intensity may vary according to indicators such as: (1) practices complexity, (2) degree of techniques specialization, (3) occurrence and types of social regulations, (4) artificial selection intensity, (5) energy invested, (6) tools types, and (7) amounts of resources obtained. Management types of edible plants were characterized and analyzed in Náhuatl communities of the Tehuacán Valley. We expected that both natural and human pressures generate risk on plant resources availability, influencing human responses of management directed to decrease risk. We particularly hypothesized that magnitude of risk would be a direct function of human pressures favored by cultural and economic value and ecological factors such as scarcity (restricted distribution and abundance). Management practices may decrease risk of plant resources, more effectively when they are more intense; however, absence or insufficiency of management practices on endangered plants may favor loss of their populations. Understanding current management motives and their consequences on the purpose of ensuring availability of plant resources might allow us to understand similar processes occurring in the past. This issue is particularly important to be studied in the Tehuacán Valley, where archaeologists documented possible scenarios motivating origins of plant management by agriculture during prehistory.

**Methods:**

Through ethnobotanical collecting, 55 semi-structured and free listing interviews we inventoried edible plant species used in five villages of Coyomeapan, Mexico. We identified: (1) native plant species whose products are obtained exclusively through simple gathering, (2) native species involving simple gathering and other management types, and (3) non-native species managed by agricultural management. We conducted in depth studies on the 33 native species managed through gathering and other types of practices. We carried out a total of 660 sessions of detailed interviews to 20 households randomly selected. We showed to people voucher specimens and photos of the sample of species chosen and documented their cultural and economic values. Spatial availability of these plant species was evaluated through vegetation sampling. Values for each cultural, economic, and ecological indicator were codified and averaged or summed and weighed according to frequency of interviewees’ responses or ecological conditions per plant species. With the standardized values of these indicators we performed a PCA and scores of the first principal component were considered as a risk index, which summarizes information of thirteen indicators of human use, demand and scarcity of each plant species. Similarly, eleven indicators of energy invested, complexity, tools and management strategies were used for performing PCA and scores of the first principal component were considered as management intensity index for each plant species. A linear regression analysis was performed to analyze the relation between risk and management intensity indexes. Amounts of variation of management data explained by ecological, cultural and economic information, as well as their risk level were analyzed through canonical correspondence analyses (CCA).

**Results:**

A total of 122 edible plant species were recorded, nearly 30% of them were introduced domesticated plants, 51 were wild species obtained exclusively by simple gathering and 33 were native species obtained by simple gathering and other management practices, these latter were the ones more deeply studied. People recognized variants in 21 of these latter 33 species, the variants receiving differential use, management, artificial selection and incipient domestication. The lowest values of management intensity corresponded to species under simple gathering and tolerance, mostly annual abundant plants, occasionally consumed by few people. The highest management intensity values were recorded in species with economic importance, mostly perennial with recognized variants whose management requires using tools, and which are protected by collective regulations. The regression analysis indicated significant value R^2^ = 0.433 (P < 0.001) between risk and management indexes. CCA explained 65.5% of variation of management intensity, mainly by socio-cultural factors (32.6%), whereas ecological data explained 21.3% and the intersection of all factors 11.6%. Variation of management intensity is 67.6% explained by risk variables. Length-span of life cycle, reproductive system type, distribution, number of parts used, number of management and use forms and type of regulations were statistically significant.

**Conclusion:**

People manage plant resources according to the role these play in households’ subsistence, the quantity available and the quality of their useful products; particularly important is the balance between resources availability and demand. Management responses to risk are also influenced by the ease to propagate or manipulate individual plants and time requiring the construction of manipulation strategies and techniques.

## Background

Humans have developed different types of interactions with their surrounding ecosystems and natural resources, continually shaping them according to their subsistence needs and other cultural purposes. Such shaping process is domestication and may involve particular resources and ecosystems. We generally define management as practices directed to transforming or adapting ecosystems, their components (e.g. natural resources) and/or its processes (e.g. ecosystem functions and services) according to human purposes [1-4]. For instance, in Mesoamerica forest management practices are commonly targeted at promoting certain compositions of vegetation, in order to ensure or increase the availability of populations of particular species, or individual phenotypes within populations which have desirable features to people [1,2,5]. Our study area is part of Mesoamerica and we were particularly interested in documenting the motives associated to such management types of forests.

The Mexican territory is highly diverse in ecosystems, species and human cultures [6]. Some authors have estimated that nearly 7,000 plant species are used by peoples in this country [7]. However, not all species are equally valued, since human groups recognize different properties and qualities of particular plant species for satisfying their needs, which influences how valuable the resources are, and such value may in turn influence how plants are managed [1-3]. In addition, peoples have developed ecological knowledge about plants they use (life cycle, distribution, abundance, interactions with other organisms) [8-10] and this information also influences the ways they interact with plants.

Human cultural values and traditional ecological knowledge of plant resources are therefore crucial for making management decisions in order to ensure or increase availability and/or quality of desired plant resources. These criteria are valid for particular species but also for particular phenotypic variants of a species [2,5]. A plant species may be managed differently in variable ecological and cultural contexts, and may involve different management intensities, degrees of specialization and complexity of practices [1,5,11-13]. To understand the motives of management and domestication of plants it is therefore helpful to analyze cultural and economic values of plant resources in relation to their spatial availability and all these factors in relation to management complexity and intensity.

Cultural importance of plant resources has been evaluated through their use frequency, amounts of products harvested or consumed, use preference, and the explicit value that people attribute to them [14-17]. Their economic importance has been evaluated through information about their exchange for other products, prices in markets, the economic value of other goods that might substitute a plant resource, or through evaluating the balance between availability and demand of products [18]. Plant resource availability has been calculated through ecological aspects such as distribution, abundance, temporal availability of useful products, adaptability to disturbed environments, length of life cycle, reproductive system type, seed dormancy or special requirements for germination, among others [3,5,19].

Several authors have proposed different criteria for characterizing and classifying plant management types [1,3,5,11,12,20-24]; most of them agree that complexity of practices and the occurrence of artificial selection are meaningful aspects that can be found in a gradient of conditions and should be the bases for developing plant management typologies [3,23,24]. In Mexico, ethnobotanical studies have documented different forms of plant management at individual, population, or community levels involving wild, weedy, and domesticated plants; such variation allows analyzing variation of management forms as well as causes of that variation. Gathering and agriculture are two main categories of human-plants interactions, but some intermediate management types or “incipient” management forms have also been documented [1,2,5,11,12,25-27]. Management practices may be carried out in the habitats where plants naturally occur (forests in the case of wild plants and human-made environments in the case of weeds), and for this reason these are called in situ management techniques. Other practices are conducted out of the natural environments of plants and for this reason are called ex situ management techniques [1,5,11,12].

Most approaches to classify management types highlight the importance of artificial selection which may vary in intensity according to the degree of systematic decisions of eliminating non-preferred phenotypes and enhancing those preferred by people, the degree of isolation of managed plant populations with respect to their wild relatives, the length of life cycle, or the type of reproductive system, among other aspects [1]. Artificial selection has been documented most commonly to occur in human made environments out of natural plant species populations. However, some studies have documented the occurrence of artificial selection in situ, associated to silvicultural practices, by selectively let standing and/or removing species or particular phenotypes in vegetation, or in association with propagation of seeds or vegetative propagules of desirable phenotypes [1-5].

We have proposed that in addition to artificial selection, other criteria used by agroecologists as indicators of agricultural management intensity [28] may also be helpful for general characterizations of plant management [3]. In a previous study we discussed a typology of plant management forms based on information on use and management of nearly 1600 useful plant species of the Tehuacán Valley [3]. We proposed that such typology should consider the following aspects: i) energy invested in practices (for instance hours and effort dedicated to management practices), use of fuel or fossil energy, use of tools or machines involving energy previously invested in producing them; ii) management strategies, planning, regulations and techniques, iii) occurrence of artificial selection and degree of intensity of this evolutionary force; and iv) production, in terms of amount or biomass of useful product per area unit. All these aspects may be indicators of creativity, measurable energy invested in practices, degree of human interest on managed plant resource, benefits obtained by practices, and degree of domestication of a plant resource. Such indicators can be observed in gradients of values from lower to higher and, accordingly, may be indicators of management intensity.

This study analyzed how such general categories of management and management intensity indicators are found in a case study: the edible plants used by Náhuatl communities of the Tehuacán Valley, central Mexico. We documented in detail all practices involved in plant management to establish more precisely than in our previous studies which indicators are relevant for classifying and constructing a precise typology. In addition, we analyzed cultural and economic values of the plant resources studied along with their abundance or scarcity in their territories. Relations of all these indicators were examined in order to identify motives of management associated to needs of ensuring or increasing plant resources availability or local people worries about plant resources availability or risk. We expected that both natural and human pressures generate risk on plant resources availability, influencing human responses of management directed to decrease risk. We particularly hypothesized that magnitude of risk would be a direct function of human pressures favored by cultural and economic value and ecological factors such as scarcity (restricted distribution and abundance). Management practices may decrease risk of plant resources, more effectively when they are more intense; however, absence or insufficiency of management practices on endangered plants may favor loss of their populations. Understanding current management motives and their consequences on the purpose of ensuring availability of plant resources might allow us to understand similar processes occurring in the past. This issue is particularly important to be studied in the Tehuacán Valley, where archaeologists documented possible scenarios motivating origins of plant management by agriculture during prehistory.

## Methods

### Study area

**Figure 1:**
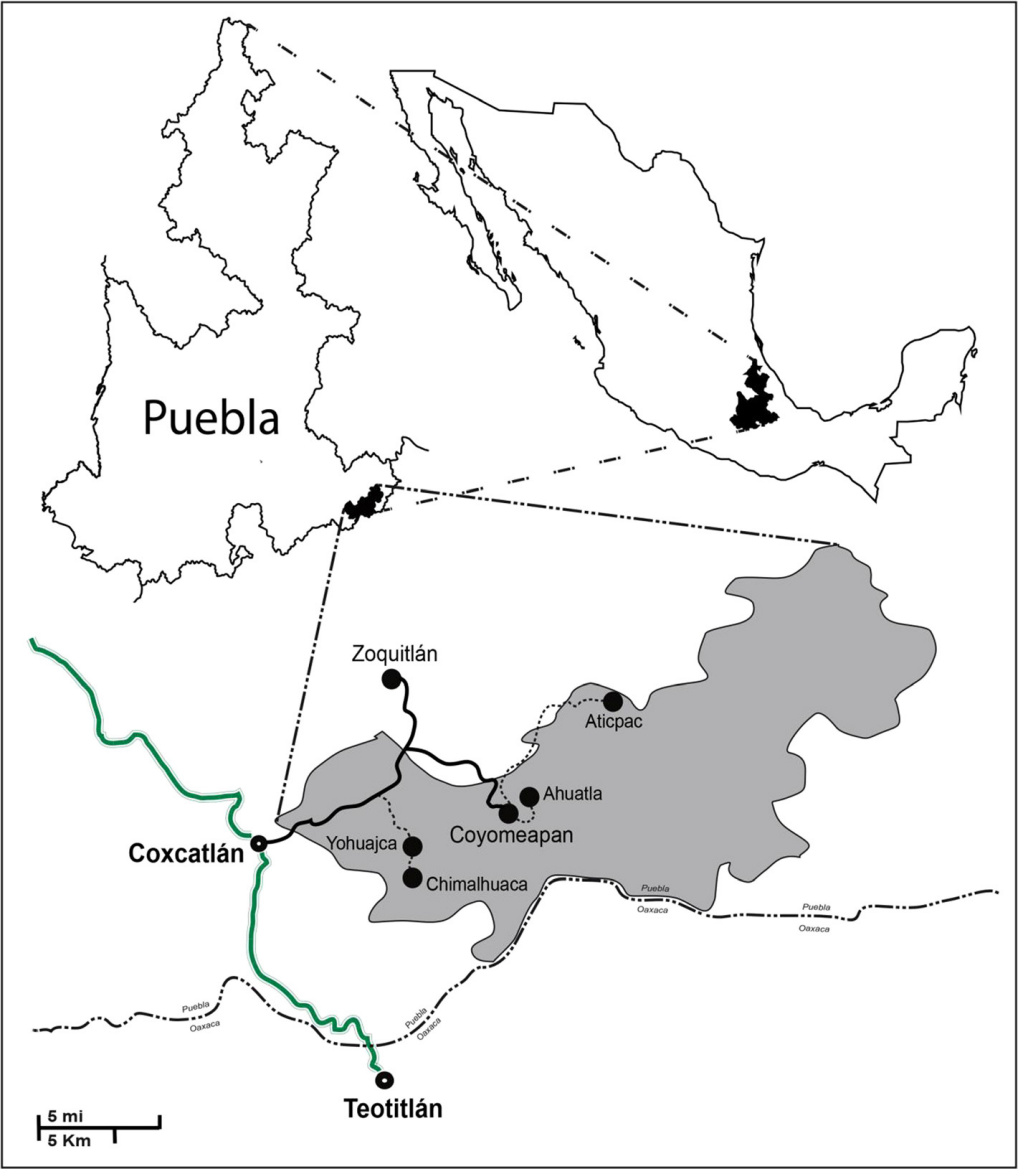
**Study area**. **Location of municipality of Santa María Coyomeapan and the villages studied in the highlands of the Tehuacán Valley, central Mexico.**

Our study was conducted in the highlands of the Tehuacán Valley, an area of high biological and cultural diversity and long history of interactions between humans and plants. We studied indigenous Náhuatl people communities in the municipality of Santa María Coyomeapan, located at the southeast of Puebla, central Mexico (Figure 1). The area belongs to the mountain range regionally known as the Sierra Negra, with elevations ranging from 1200 to 3250 m. Annual mean temperature and precipitation are on average 16°C and 2200 mm, respectively [29]. The region hosts the following vegetation types: I) Pine-oak forest, II) evergreen rainforest and III) tropical dry forest. Coyomeapan is a rural region where people’s subsistence mainly depends on agriculture, raising of domestic animals, and extraction of forest products. Nearly 98% of the 12,000 people inhabiting the area are Náhuatl people. Part of the local households also obtains incomes from annual seasonal migration to cities in Mexico and the United States.

### Inventory of edible plant resources

During a first year of field work we inventoried the edible plant resources in five communities: Aticpac, Ahuatla, Chimalhuaca, Yohuajca and Coyomeapan (Figure 1). For inventorying all plant species used as food we interviewed a total of 55 household selected at random in the villages studied. Through semi-structured interviews [30], we obtained information about plant uses, management forms, ethnoclassification of variants, social and cultural roles of plants, and ecological data on distribution, abundance, phenology, and biotic interactions (Additional file 1). We used interviews with the free listing method as a first approach to identify main plant resources perceived by people. We sampled and collected plant specimens in natural vegetation, markets, crop fields, and homegardens. This information was complemented with direct observations. Plant specimens were deposited in the National Herbarium of Mexico (MEXU).

### Detailed in-depth interviews

Additional information was obtained during a second year of fieldwork through in-depth interviews aimed at documenting detailed data about the 33 native edible plant species that people managed in some way additional to simple gathering. These interviews explored information on ecological (perceived abundance, distribution and temporal availability) and sociocultural aspects (use of inputs, extraction rates, harvesting and consumption frequencies, use of tools, labor force invested, economic value, labor maintenance, methods of artificial selection) of these native species. To obtain this information, voucher specimens and photos of the 33 species referred to were shown to a random sample of 20 households (Table 1). Each person was asked to answer a total of 36 questions (Tables 2, 3, 4 and 5). We completed the interviews in nearly 660 sessions of approximately 2 hours each in average.

**Table 1:** **Edible plant species managed in Santa María Coyomeapan**, **Puebla**

**Species**	**Common name**	^**1 **^**Useful parts (Mostly)**	^**2 **^**Availability**	^**3 **^**Forms of propagation**	^**4 **^**Economic importance**
*Agave obscura* Schiede	Mexcalli cacaya	RP	S	VP, TI	CT, Ex
*Agave salmiana* Otto ex Salm-Dyck	Mexcalli mateuonti	CI	S	VP, TI	CT
*Amaranthus hybridus* L.	Baquilitl	VP	S	S	CT
*Brassica rapa* L.	Colesh	VP	S	S	*
*Canna indica* L.	Panispatl	VP	C	TI, S	*
*Cestrum nocturnum* L.	Zopelilquilitl	VP	C	VP	CT, Ex
*Chamaedorea tepejilote* Liebm. ex Mart	Tepejilote	CI	S	TI, S	CT, Ex
*Cleoserrata speciosa* (Raf.) H.H. Iltis	Mabilquilitl	VP	S	S	CT
*Crataegus mexicana* Moc. & Sessé ex DC.	Xocotebitl	CI	S	TI	Ex
*Dasylirion serratifolium* (Karw. ex Schult. f.) Zucc.	Mazitzi	RP	S	TI	CT, Ex
*Eugenia capuli* (Schltdl. & Cham.) Hook. & Arn.	Mototetl	CI	S	TI	CT, Ex
*Inga vera* Kunth	Topetli	RP	S	TI	*
*Jatropha curcas* L.	Piñòn	RP	S	VP	CT
*Leucaena leucocephala* (Lam.)	Baxi	RP	S	TI, S	CT, Ex
*Litsea glaucescens* Kunth	Sogogotl	VP	C	TI	CT, Ex
*Peperomia peltilimba* C. DC.	Tequilitl	CI	C	VP	CT, Ex
*Phaseolus coccineus* L. (Nezoquilitl)	Xochiquilitl	RP	S	S	Ex
*Phytolacca icosandra* L.	Molquilitl	VP	C	S	*
*Piper auritum* Kunth	Tlanilpaquilitl	VP	S	VP, TI	*
*Plantago alismatifolia* Pilg.	Toro lengua	VP	S	S	*
*Porophyllum ruderale* (Jacq.) Cass.	Pipicha	CI	C	S	CT, Ex
*Prunus serotina* Ehrh.	Capulli	RP	S	TI, S	CT, Ex
*Quercus candicans* Née	Tamalabatl	VP	C	TI	*
*Raphanus raphanistrum* L.	Rábano	VP	S	S	*
*Renealmia alpinia* (Rottb.) Maas	Velijmolli	VP	C	TI, VP, S	CT, Ex
*Sambucus mexicana* C. Presl ex DC.	Xometl	VP	C	VP	*
*Sideroxylon palmeri* (Rose) T.D. Penn.	Tempesquistle	RP	S	TI	CT, Ex
*Solanum americanum* Mill.	Tomaquilitl	VP	S	S	Ex
*Sonchus oleraceus* L.	Memella	VP	S	S	*
*Spathiphyllum cochlearispathum* (Liebm.) Engl.	Elotlquilitl	RP	S	TI, VP	CT, Ex
*Tigridia pavonia* (L. f.) DC.	Tlalteztli	VP	C	S	*
*Vaccinium leucanthum* Schltdl.	Tetzmolli	RP	S	TI	*
*Yucca elephantipes* Regel	Izotl	RP	S	VP	CT, Ex

^1^ Mostly useful parts - *CI* Complete Individuals, *RP* Reproductive parts, Vegetative parts.

^2^ Disponibility - *S* Seasonally, *C* Continual.

^3^ Forms of propagation- *S* Seed; *TI* Transplantation Individuals; *VP* Vegetative parts.

^4^ Economic Importance - *CT* Cash transaction; *Ex* Exchanged; * Without economic importance.

**Table 2:** **Variables considered for analyzing management intensity** (**numbers in parentheses are the codified values**)

**Variable**	**States of variables and codified values**										
Lifecycle	Perennial (1)					Annual (2)					
Method of reproduction	Sexual (1)			Asexual (2)			Sexual and Asexual (3)				
Reproductive System	Mostly self-incompatible (1)					Mostly self-compatible (2)					
Maintenance Labours	Cleaningor weeding (0.5)	Grooves water penetration (0.5)	Softening soil (0.5)	Remove dead leaves or pruning branches (0.5)	Apply fertilizer (0.5)	Irrigation (0.5)	Fumigation (0.5)	Calcimine (0.5)	Separate clumps or propagules (0.5)	Place of guardians rods (0.5)	Make fences (0.5)
Artificial selection	Odor (1)	Form (1)	Color (1)	Flavor (1)	Size(1)	Phenological differences (1)			Texture (1)		
Collective regulation	No regulation (0)		Yes, but does not apply (1)			Yes, admonition applies (1.5)			Yes, applies monetary penalty (2)		
Using Tools	Manual (0.5)	Stem, pole or equivalent (1)		Knife, penknife or equivalent (1.5)		Machete, sickle or equivalent (2)		Axe, shovel or equivalent (2.5)		Specialized (3)	
Proximity to site collects	Less than 100 mg (0.5)		Up to 1 Km (1)			Up to 5 km (1.5)			More than 5 km (2)		
Time spent in collecting	Minutes (0.5)				Hours (1)			Days (2)			
Management forms	Simple collection (0.5)	Regulated collection (1)	Tolerance (1.5)	Enhancement (2)	Protection (2.5)	Vegetative parts transplantation (3)			Transplantation individuals (3.5)		Sowing seeds (4)

**Table 3:** **Ecological**, **sociocultural and economic parameters calculated for the 33 edible species considered in this study**

**Species**	**Cultural importance**	**Economic importance**	**Abundance perceived**	**Density indiv/ha**	Biomass **cover%	**Ecological dominance index**	**Distribution %**
*Chamaedorea tepejilote* Liebm. ex Mart	0.2919	^(5)^ 0.227	^(4)^ 1.920	^(3)^ 1166.666	^(5)^ 1310.965	^P(3)^ 2016995.962	33.333
*Agave salmiana* Otto ex Salm-Dyck	0.2427	^(2)^ 0.303	^(20)^ 3.000	0.000	0.000	^P^ 0.000	0.000
*Litsea glaucescens* Kunth	0.1608	^(1)^ 0.394	^(12)^ 2.550	^(12)^ 20.000	^(12)^ 15.241	^P(12)^ 60.967	33.333
*Peperomia peltilimba* C. DC.	0.1103	^(10)^ 0.133	^(17)^ 2.800	-	**^(8)^ 0.086	^A(7)^ 0.087	11.111
*Leucaena leucocephala* (Lam.) de Wit	0.0965	^(19)^ 0.034	^(28)^ 3.750	0.000	0.000	^P^ 0.000	0.000
*Eugenia capuli* (Schltdl. & Cham.) Hook. & Arn.	0.0869	^(6)^ 0.163	^(31)^ 4.000	^(11)^ 20.000	^(10)^ 129.520	^P^ 0.000	11.111
*Yucca elephantipes* Regel	0.0408	^(12)^ 0.080	^(18)^ 2.880	^(6)^ 146.666	^(3)^ 5501.371	^P(5)^ 225594.800	22.222
*Prunus serotina* Ehrh.	0.0357	^(15)^ 0.062	^(9)^ 2.140	^(9)^ 80.000	^(6)^ 1146.264	^P(8)^ 18340.231	11.111
*Piper auritum* Kunth	0.0319	* 0.001	^(22)^ 3.000	-	**^(3)^ 0.208	^P(11)^ 518.083	0.000
*Porophyllum ruderale* (Jacq.) Cass.	0.0316	^(3)^ 0.278	^(10)^ 2.220	-	**^(9)^ 0.086	^A(8)^ 0.087	11.111
*Canna indica* L.	0.0283	* 0.001	^(8)^ 2.100	^(7)^ 140.000	^(11)^ 55.334	^P(10)^ 1549.356	11.111
*Agave obscura* Schiede	0.0234	^(11)^ 0.085	^(11)^ 2.430	0.000	0.000	^P^ 0.000	0.000
*Amaranthus hybridus* L.	0.0215	^(4)^ 0.259	^(3)^ 1.730	-	**^(4)^ 0.185	^A(3^ 0.185	22.222
*Brassica rapa* L.	0.0171	* 0.001	^(16)^ 2.670	-	**^(7)^ 0.162	^A(6)^ 0.162	22.222
*Plantago alismatifolia* Pilg.	0.0136	* 0.001	^(29)^ 3.750	-	**^(12)^ 0.075	^A(11)^ 0.075	11.111
*Cestrum nocturnum* L.	0.0133	^(9)^ 0.139	^(19)^ 2.900	^(4)^ 480.000	^(7)^ 548.163	^P(6)^ 210494.830	22.222
*Crataegus mexicana* Moc. & Sessé ex DC.	0.0128	^(14)^ 0.064	^(33)^ 4.090	0.000	0.000	^P^ 0.000	0.000
*Solanum americanum* Mill.	0.0092	^(20)^ 0.025	^(14)^ 2.600	-	**^(5)^ 0.185	^A(4)^ 0.185	22.222
*Phaseolus coccineus* L.	0.0075	^(18)^ 0.050	^(23)^ 3.100	-	**^(10)^ 0.075	^A(9)^ 0.075	11.111
*Quercus candicans* Née	0.0070	* 0.001	^(1)^ 1.560	^(1)^ 5180.000	^(1)^ 36436.946	^P(1)^ 169869044.816	33.333
*Dasylirion serratifolium* (Karw. ex Schult. f.) Zucc.	0.0046	^(7)^ 0.156	^(5)^ 2.000	^(5)^ 260.000	^(8)^ 454.600	^P(7)^ 94556.988	11.111
*Phytolacca icosandra* L.	0.0032	* 0.001	^(15)^ 2.640	-	**^(11)^ 0.075	^A(10)^ 0.075	11.111
*Renealmia alpinia* (Rottb.) Maas	0.0024	^(13)^ 0.070	^(25)^ 3.330	-	0.000	^P^ 0.000	0.000
*Sambucus mexicana* C. Presl ex DC.	0.0016	* 0.001	^(2)^ 1.710	^(10)^ 60.000	^(9)^ 176.668	^P(9)^ 2120.022	0.000
*Sideroxylon palmeri* (Rose) T.D. Penn.	0.0013	^(8)^ 0.148	^(27)^ 3.500	0.000	0.000	^P^ 0.000	0.000
*Inga vera* Willd.	0.0008	* 0.001	^(24)^ 3.290	^(8)^ 90.000	^(2)^ 7462.929	^P(4)^ 1041733.572	33.333
*Spathiphyllum cochlearispathum* (Liebm.) Engl.	0.0008	^(16)^ 0.060	^(32)^ 4.000	-	**^(2)^ 0.231	^A(2)^ 0.231	22.222
*Jatropha curcas* L.	0.0006	^(17)^ 0.053	^(21)^ 3.000	0.000	0.000	^P^ 0.000	0.000
*Sonchus oleraceus* L.	0.0004	* 0.001	^(13)^ 2.570	-	**^(6)^ 0.185	^A(5)^ 0.185	22.222
*Cleoserrata speciosa* (Raf.) H.H. Iltis	0.0001	^(21)^ 0.021	^(30)^ 3.880	-	0.000	^A^ 0.000	0.000
*Raphanus raphanistrum* L.	0.0001	* 0.001	^(6)^ 2.000	-	**^(13)^ 0.075	^A^ 0.000	0.000
*Vaccinium leucanthum* Schltdl.	0.0001	* 0.001	^(7)^ 2.000	^(2)^ 1920.000	^(4)^ 1897.257	^P(2)^ 3642734.885	33.333
*Tigridia pavonia* (L. f.) DC.	0.0000	* 0.001	^(26)^ 3.440	-	**^(1)^ 0.295	^A(1)^ 0.295	33.333

*Species without economic importance.

( ) The number in parentheses indicates the hierarchical order of each species in the calculated parameters.

**Percentage of cover for annual species.

*P* Perennial species, *A* Annual species.

**Table 4:** **Matrices used in the partial canonical ordination** (**CCA**)

**Matrices**	**Variables**	**Description**
Management Intensity (Response Matrix Y)	Life cycle	Annual or perennial.
	Method of reproduction	Sexual, asexual or both.
	Reproductive System	Mostly self-incompatible or Mostly self-compatible.
	Maintenance Labours	Cleaning or weeding, grooves water penetration, apply fertilizer, fumigation, etc.
	Artificial selection	If selection criteria are recognized by specific characteristic.
	Collective regulation	Existence of rules governing access to the resource and how it is applied.
	Using Tools	Types of tools used in resource extraction.
	Proximity to site collects	Distance in meters from households to the extraction sites.
	Time spent in collecting	Minutes, hours, and days.
	Management types	Conditions of a plant’s management, whether gathered, tolerated, promoted, protected or cultivated.
Ecological (Matrix X)	Spatial distribution	Percentage of plots in which each species is present.
	Temporal distribution	Harvested parts are available continuously throughout the year or only seasonally.
	Lyfe cycle	Annual or perennial.
	Reproductive System	Mostly self-incompatible or Mostly self-compatible.
	Ecological Dominance Index	Value calculated from the frequency, biomass, coverage and density.
	Usefulparts	Mostly vegetative parts, mostly reproductive parts or whole individuals.
	Frecuency	Proportion of presence in the quadrants of each sampling.
	Cover	Percentage of cover in three quadrants of 1m^2^ for annuals.
	Biomass	Calculated from the hedges and the diameter at breast height for perennials.
	Density	Number of individuals per hectare.
Sociocultural and economic (Matrix W)	Consumption	Number of people consuming any edible species considered in this study.
	Frequency of use	Consumption over the year.
	Last day of consumption	Days, weeks, months or years.
	Uses	Number of uses that have a species.
	Useful parts	Mostly vegetative parts, mostly reproductive parts or whole individuals.
	NumberUsefulParts	Total number useful parts.
	Commercialization	Local market presence.
	Medicinal use	Medicinal use edible addition.
	Average price	Average price of a plant species in all markets.
	Sales Volume	Total sales volume in local market.
	People who sell	People in the community who market some resource.

**Table 5:** Indicators and the numerical values assigned for analyzing risk of edible plant species

**Variables**	**Scale**
Life cycle	Annual (1); Perennial (2)
Reproductive System	Mostly self-compatible (1); Mostly self-incompatible (2)
Distribution	Broad (1); Restricted (2)
Abundance perceived	Very abundant (1); Abundant (2;) Regular abundance (3); Escarse (4); Very escarse (5)
Useful parts	Mostly vegetative parts (1); Mostly reproductive parts (2); Complete individuals (3)
Availability	Continuous (1); Temporal (2)
Plagues	No pest (1); Presents pests, but nothing is done to eliminate them (2); Presents pests and these are eliminated (3)
Number of used parts	Number of parts utilized
Management	With management (1); Without management (2)
Norms of use	No rule (1); With rule, but this does not apply (2); With rule, and this are applied (3)
Cultural Importance	Value calculated for Cultural Importance Index
Economic Importance	Value calculated for Economic Importance Index
Distribution	In over 30% of plots (0.5); Up to 20% of plots (1); Up to 10% of plots (1.5); Not found in the plots (2)

We constructed matrixes with the responses of each person interviewed codified as explained in Tables 2, 3, 4, 5, 6 and 7 for each plant species. Also, the value of the responses were averaged or summed and weighed according to the frequency of the responses considering the whole sample of interviewees. The values per species were used for calculating the indexes explained below.

**Table 6:** Parameters and values used for estimating the management intensity index

**Species**	**Lifecycle**	**Method of reproduction**	**Reproductive system**	**Maintenance**	**Artificial selection**	**Collective regulation**	**Using tools**	**Proximity to collecting sites**	**Time spent in collecting**	**Management forms**	**Management intensity index**
*Agave obscura* Schiede	1	3	1	0.167	0.000	0.000	0.533	0.733	0.433	1.233	8.100
*Agave salmiana* Otto ex Salm-Dyck	1	3	2	0.467	0.000	0.667	1.633	0.467	0.467	1.900	11.600
*Amaranthus hybridus* L.	2	1	1	0.333	0.800	0.000	0.400	0.600	0.367	2.367	8.867
*Brassica rapa* L.	2	1	1	0.267	0.800	0.000	0.100	0.500	0.333	1.867	7.867
*Canna indica* L.	1	3	2	0.333	0.733	0.000	0.633	0.233	0.300	2.233	10.467
*Cestrum nocturnum* L.	1	2	2	0.267	0.600	0.000	0.400	0.400	0.267	2.067	9.000
*Chamaedorea tepejilote* Liebm. ex Mart	1	3	1	0.533	0.933	0.267	1.167	1.267	0.800	4.200	14.167
*Cleoserrata speciosa* (Raf.) H.H. Iltis	2	1	2	0.167	0.000	0.000	0.233	0.133	0.133	1.233	6.900
*Crataegus mexicana* Moc. & Sessé ex DC.	1	2	1	0.367	0.133	0.133	0.133	0.700	0.333	2.333	8.133
*Dasylirion serratifolium* (Karw. ex Schult. f.) Zucc.	1	2	1	0.300	0.400	0.867	0.667	1.467	0.267	0.767	8.733
*Eugenia capuli* (Schltdl. & Cham.) Hook. & Arn.	1	1	1	0.333	0.000	0.000	0.533	1.067	0.800	3.000	8.733
*Inga vera* Willd.	1	3	1	0.267	0.200	0.000	0.533	0.200	0.133	2.000	8.333
*Jatropha curcas* L.	1	2	2	0.233	0.000	0.000	0.267	0.100	0.100	1.900	7.600
*Leucaena leucocephala* (Lam.) de Wit	1	3	2	0.433	0.600	0.067	0.333	0.733	0.233	3.300	11.700
*Litsea glaucescens* Kunth	1	1	1	0.300	0.400	1.000	0.400	1.167	0.800	2.800	9.867
*Peperomia peltilimba* C. DC.	1	2	2	0.167	0.267	0.000	0.267	0.900	0.500	0.700	7.800
*Phaseolus coccineus* L.	1	3	2	0.400	0.000	0.000	0.300	0.367	0.333	1.833	9.233
*Phytolacca icosandra* L.	1	1	1	0.067	0.000	0.000	0.100	0.400	0.300	0.533	4.400
*Piper auritum* Kunth	1	2	2	0.400	0.467	0.000	0.233	0.500	0.333	2.000	8.933
*Plantago alismatifolia* Pilg.	2	1	1	0.200	0.600	0.000	0.300	0.600	0.333	1.067	7.100
*Porophyllum ruderale* (Jacq.) Cass.	2	1	1	0.400	0.267	0.000	0.233	0.933	0.367	2.800	9.000
*Prunus serotina* Ehrh.	1	3	1	0.333	0.600	0.667	0.333	0.333	0.233	2.967	10.467
*Quercus candicans* Née	1	3	2	0.367	0.600	0.867	1.067	1.000	0.533	1.367	11.800
*Raphanus raphanistrum* L.	2	1	2	0.133	0.200	0.000	0.200	0.200	0.100	0.700	6.533
*Renealmia alpinia* (Rottb.) Maas	1	3	1	0.300	0.400	0.000	0.600	0.200	0.133	1.900	8.533
*Sambucus mexicana* C. Presl ex DC.	1	3	1	0.167	0.000	0.000	0.333	0.167	0.167	0.933	6.767
*Sideroxylon palmeri* (Rose) T.D. Penn.	1	1	1	0.200	0.200	0.000	0.267	0.133	0.167	1.000	4.967
*Solanum americanum* Mill.	2	1	1	0.133	0.467	0.000	0.200	0.600	0.333	1.133	6.867
*Sonchus oleraceus* L.	2	1	1	0.100	0.267	0.000	0.133	0.333	0.167	0.900	5.900
*Spathiphyllum cochlearispathum* (Liebm.) Engl.	1	2	2	0.100	0.467	0.000	0.600	0.233	0.200	1.133	7.733
*Tigridia pavonia* (L. f.) DC.	1	1	2	0.067	0.000	0.000	0.467	0.200	0.133	0.500	5.367
*Vaccinium leucanthum* Schltdl.	1	1	1	0.200	0.000	0.000	0.200	0.567	0.333	0.933	5.233
*Yucca elephantipes* Regel	1	2	1	0.300	0.000	0.000	0.933	0.400	0.300	1.400	7.333

**Table 7:** Recognition of variants in species with management in Coyomeapan

***Species***	***Object of selection***	***Characteristics of the recognized variants***	***Preferred variant***
*Amaranthus hybridus* L.	Leaves	1. White: inflorescence with white seeds and clear green leaves.	White. Since it has a more delicate flavor. The other variants are more bitter.
		2. Purple: inflorescence with red seeds and leaves with purple edges.	
		3. “Pinto” (spotted): Inflorescence reddish leaves with purple spots.	
*Brassica rapa* L.	Entire plant	2. “Colesh”: stem clean and smooth, pleasant taste.	Colesh. Since it has soft leaves that are easy to digest.
		3. “Coleshteneztli” or “Cashtelanquilitl” (Colesh goat): stem tomentose, scratchy texture, bitter leaves.	
*Canna indica* L.	Entire plant	1. “Panisplatl de tamal”: Flowers small, long leaves and light green.	Both are appreciated, but they serve and are propagated with different purposes.
		2. “Panispatl ornament”: Flowers large and showy; leaves medium gray-green.	
*Cestrum nocturnum* L.	Young leaves	1. Leaves with pleasant flavor when cooked. Flowering very conspicuous.	Leaves with pleasant flavor. The bitter variety is an emerging food.
		2. Leaves with bitter flavor. Rarely blooms. It is known as wild.	
*Chamaedorea tepejilote* Liebm. ex Mart.	Male inflorescence immature	1. “Tepejilote Metlapilli”: Inflorescence large and thick.	The first three are prized for their yield and their market price. The latter is a emerging food.
		2. “Tepejilote tronquitos”: Inflorescence small and thick.	
		3. “Tepejilote of plantation coffee”: Inflorescence of size and average.	
		4. thickness, but high productivity.	
		5. Tepejilote “Corpus” (wild): Inflorescence small and thin.	
*Dasylirion serratifolium* (Karw. ex Schult. f.) Zucc.	Young inflorescence	1. Inflorescence purple, and flower buds larger.	Inflorescence purple. Because it has higher yield and better price. However, both varieties are sold.
		2. Inflorescence white, and bud smaller.	
*Inga vera* Willd.	Leaves	1. “Topetli of plantation coffee”: large leaves.	The variety of coffee plantation, since it is used to shade coffee.
		2. “Topetli wild”: Small leaves and edible fruit, but not sown.	
*Leucaena leucocephala* (Lam.)	Seeds	1. White: Pods green clear and pleasant taste.	With the exception of the variety "prieto", all others are consumed with no clear preferences.
		2. Red: dark green leaves and more concentrated flavor.	
		3. Pink: sweeter taste.	
		4. “Prieto”: Variety with bark dark, recognized as wild.	
*Litsea glaucescens* Kunth	Leaves	1. “Laurel of odor”: leaves thin and small, grayish underside. Tiny flowers.	“Laurel of odor”. He is recognized as "authentic". Best flavor food and therefore has the best price on the market.
		2. Leaves broad and elongated, light green undersides. Larger flowers.	
*Peperomia peltilimba* C. DC.	Leaves	1. “Tequilitl”: Small leaves, thin, and smooth taste.	Tequilitl.It is recognized as edible and is sold in the market. Tehuantequilitl not sold and is recognized as ornamental.
		2. “Tehuantequilitl” (quelite of coyote): Larger leaves and thicker. Flavor more concentrated.	
*Piper auritum* Kunth	Leaves	1. “Tlanilpaquilitl of house”: Leaves large, smooth, and highly aromatic. Smooth stems and light green.	“Tlanilpaquilitl of house”. Its leaves are sweet and fragrant.
		2.“Tlanilpaquilitl wild”: Leaves small and odorless. Stems with white spots.	
*Plantago alismatifolia* Pilg.	Leaves	1. “Nenepilpitzabatl”: Leaves thin and elongated.	“Nenepilpitzabatl”. Since it has soft leaves, which are very easy to digest. The other varieties have leaves rough and rugged.
		2. Variety of broad-leaved. It is used to feed the turkeys.	
		3. Wild: Leaves reddish and bitter taste, not eaten.	
*Porophyllum ruderale* (Jacq.)Cass.	Complete plant	1. White: Light green leaves. Cultivated.	Both are appreciated. Although the "white" is cultivated by irrigation, which guarantees to be present throughout almost all year. By contrast, the seed of the variety "purple" is spread in the fields of temporal.
		2. Purple: Leaves and flowers of purple coloration. This grows in the dry zone.	
*Prunus serotina* Ehrh.	Fruit	1. “Capulín of house”. Red fruits, sweet taste, light green leaves.	Capulín of house.This is sold in local and regional markets. The variety “capulín of fox" the fruits are not consumed. The wood is used as firewood and for making tools.
		2. “Capulli Iztotzi or Capulli Quimichi” (Capulín of fox or of mouse). Black fruit with acid flavor, dark green leaves.	
*Quercus candicans* Née	Leaves	1. Leaves large, thin, smooth. Leaf underside glabrous and bcenter. The surface of the leaf is light green.	Leaf smooth and glabrous. Can be handled better.
		2. Leaves thick, leathery, spoon-shaped. Undersides densely tomentose. Beam darker color.	
*Raphanus raphanistrum* L.	Leaves	1. Pubescent leaf, purple flower, intense flavor and hard to digest.	Smooth leaf. It is more digestible. The purple variety is recognized as fodder.
		2. Smooth leaf, white flower and sweeter taste.	
*Renealmia alpinia (Rottb.)* Maas	Complete plant	1. “Velijmolli”: Dark green leaves with slightly wavy edges. Rounded tips.	Velijmolli. They have larger leaves, higher performance, and better price.
		2. “Velijmolli wild”. Leaves light green, smooth edge. Ending in a peak.	
*Sideroxylon palmeri* (Rose) T.D. Penn.	Fruit	1. Fruit round.	Locally are consumed the two varieties. However, the variety of round fruit at regional sells better.
		2. Fruit shaped-avocad.	
*Solanum americanum* Mill.	Complete plant	1. Bitter or wild.	The non-bitter. However, the bitter has been identified as *Solanum nigrescens* Mart. & Gal.
		2. Not bitter.	
*Sonchus oleraceus* L.	Leaves	1. White: Stems light green. Broad leaves.	White and purple are recognized as edibles. The variety green is recognized as wild and just is consumed as food emerging.
		2. Purple: Stems with purple beam.	
		3. Green: Stems green. Thin leaves, ending in a peak. Bitter taste.	
*Spathiphyllum cochlearispathum* (Liebm.) Engl.	Inflorescence	1. “Iztacxóchitl”: White flower, is not edible, bitter, is used for adornment, wild plant.	Elotlxóchitl. Its are better flavor and is sold in the market.
		2. “Elotlxóchitl or Oloxóchitl”. Green flower. Fast cooking and palatable.	

### Management intensity index

We calculated a management intensity index summarizing information of eleven indicators related to energy invested in management, type of tools used, complexity of regulations and institutions, artificial selection intensity and biological aspects influencing rapidness of management results (Table 2). Indicators used were: MF = Management form (or forms according to the general types mentioned above); IT = Invested Time (time invested in managing plants or obtaining plant products); PSD = Plant Site Distance (distance from town to sites where plant resources are found; T = Tools (type of tools used in management); CR = Collective Regulations (rules, agreements for accessing and protecting plant resources); AS = Artificial Selection (occurrence of selection criteria and type of selection practiced); ML = Maintenance Labors (type of activities carried out for ensuring availability of plant resources); RS = Reproductive System Type; MR = Modes of Reproduction, and LC = Life Cycle type. The standardized values of these indicators were used to perform a Principal Component Analyses by NTSYS. Scores of the first principal component were used as management intensity indexes, which were calculated for the 33 native managed species.

### Cultural and economic values of edible plant species

The index of cultural value (Ic) of the 33 native managed edible species was calculated based on indexes previously designed by Pieroni and González-Insuasti et al. [24,31]. Our modified index was:

$$\mathrm{Ic}=\frac{\mathrm{PULdcNuSNsuHtCM}}{10000}$$

Where: P = number of sampled persons who ate the species; U = use frequency (once or less per year = 1; up to twice a year = 2; up to 10 times a year = 3; more than 10 times a year = 4; more than once a week = 5); Ldc = last day of consumption (more than one year ago = 1; less than one year ago = 2; less than 6 months ago = 3; within the last month = 4; within the last week = 5; Nu = Number of different uses; S = structures used as food (mostly vegetative parts / leaves, branches = 1; Mostly reproductive parts / flowers, seeds, fruits = 2; complete individual plants = 3); Nsu = Number of structures consumed; Ht = Harvest type (opportunistic = 1; dedicated = 2); C = commercialization possibilities (non-existing = 1; existing = 2); M = medicinal use (not medicinal = 1; edible plants considered also as medicine = 2).

The economic value was calculated for the 33 species referred to above considering the amount of plant products per species that is commercialized, the standardized price per kg or liter in the local market, and the proportion of respondents who commercialize products of a plant species. Since plant products are commercialized in different units, we standardized amounts of products to one kg or liter and prices in U.S. dollars. We used the formula:

Ev=PQN

in which Ev = economic value; P = price per kilogram or liter of product (for species not commercialized we assigned an arbitrary value of $0.001 U.S. dollars); Q = amount of product per species annually marketed, and N = proportion of respondents who commercialize a plant species product (Table 3).

### Distribution and abundance of plant resources

Plant communities of natural environmental (i. e. non-agricultural or urban) units were sampled in three sites representing each vegetation type: tropical dry forest, pine-oak forest and tropical rain forest. In addition, artificially transformed units were sampled, including areas of secondary vegetation, fruit plantations and maize fields. Vegetation sampling was conducted through 500 m^2^ rectangles of 50x10 m (see Valiente-Banuet et al. [32]). Shrubs and trees were counted and the density of each species calculated. Biomass of each species was calculated based on measurements of height (h) and two diameters (D1 and D2) of the canopy of every individual of shrubs and trees included in the 500 m^2^ samples. Diameter at breast height (d) was also considered for sampled trees. Biomass estimations were conducted by using volume formulas of geometric figures resembling the physiognomy of the plant species recorded [33]. For herbs, we used three 1 m^2^ squares randomly distributed in each 500 m^2^ rectangle and biomass was calculated as the cover percentage per m^2^.

Distribution of edible plant species was evaluated as the percentage of all sites sampled where each species was recorded (Table 3). We also documented the perception of abundance of plant resources by local people through an index of scarcity. We used five images with the form of a star which were showed by the researchers to stimulating responses of people interviewed. The image showing 100% of colored cover was the category very abundant (value 1), that with 80% was the category abundant (2), that with 60% was the category moderately abundant (3), that with 40% was the category scarce (4) and that with 20% of colored cover was the category very scarce (5). Table 3 summarizes average abundance value perceived by people for the 33 species analyzed.

### Risk index

For evaluating risk of edible plant species we considered ecological and sociocultural variables for which higher values indicated higher risk (Table 5), as well as management intensity. For each species the risk value was the first principal component of a total of thirteen indicators. We standardized values of this index to a scale from 0 to 1, the maximum risk value being 1.

### Data analyses

a) Variation partitioning of management

**Figure 2:**
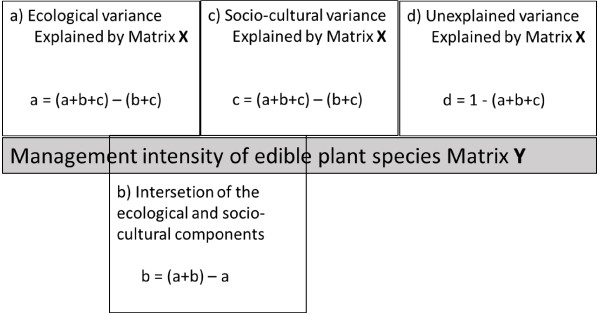
**Influences (pure and combined) of ecological (matrix X) and sociocultural (matrix W) explanatory variables on management of edible species (matrix Y).** Each CCA model involves different subsets of variation sources. For instance, matrix X affects matrix Y (Y~X) but in X coexists intermingled variation sources a) and b). Modified from Boccard et al. [36]. **a**) Ecological fraction of management intensity variation that can be explained only by ecological data, **b**) Sociocultural + ecological data, **c**) Sociocultural fraction of management intensity variation explained only by sociocultural data, and **d**) Undetermined data or fraction of management intensity variation explained neither by ecological nor by sociocultural data.

In these analyses and others explained below we centred our attention on the 33 native edible plant species receiving a management type. Canonical Correspondence Analyses (CCA) were performed to measure the amount of variation of management data explained from ecological and sociocultural information. The analyses were conducted using the R software [34,35]. Based on Boccard et al. [36] we used three matrices partitioning the variation: Matrix Y containing the response variables (management intensity data matrix), matrix X with the set of explanatory ecological variables; and matrix W with the set of explanatory sociocultural variables (Table 4, Figure 2). The main purpose of this analysis is to cope with the confounding effects that may occur if a CCA of Y is made using W or X as the only explanatory matrix. That is, some variables of W may influence variables of X and vice versa. Through this method we conducted several CCA combining sets of explanatory variables: 1) Correspondence Analysis (CA) only for matrix Y, 2) CCA for matrix Y vs. matrix W, 3) CCA for matrix Y vs. matrix X, 4) CCA for matrix Y vs. matrices W+X. The total constrained eigenvalue of each analysis was tallied to identify how much of the management intensity matrix is explained by ecologic and sociocultural data. This method allowed dividing CCA variation into four parts: a) Ecological data, which is the fraction of management intensity variation that can be explained by ecological data independently of sociocultural data, b) Sociocultural + ecological data, c) Sociocultural data which is the fraction of management intensity variation that can be explained by sociocultural data independently of ecologic data, and, d) Undetermined data or fraction of management intensity variation explained neither by ecological nor by sociocultural data (Figure 2). For each of these analyses, the sum of all canonical eigenvalues divided by the sum of all canonical eigenvalues, allowed calculating the corresponding fraction of variation explained by the analysis. Significance of the models for each CCA was estimated by permutation tests for: a) the whole model, b) management intensity explained by ecological variables and 3) management intensity explained by sociocultural variables.

b) Management as response of risk

To explore how management is a response to risk we performed a canonical correspondence analysis (CCA) with a data matrix with information on management intensity and the other with the risk index indicators (Tables 3 and 6).

## Results

### Inventory of edible plant resources

A total of 122 edible plant species were recorded, nearly 67% of them were native to the region and the remaining species were introduced from other areas (Additional file 1). These species belong to 50 botanical families, Rosaceae and Solanaceae provide the greatest number of species (10 each); followed by Fabaceae (8); Cucurbitaceae (7); Asteraceae (6) and Agavaceae (5). A total of 55 species (45%) are domesticated plants, and 35 (28.6%) are wild species obtained exclusively from simple gathering. We identified 33 species (27%) of wild plant species under one or more management types. In addition, we recorded 23 species of edible ruderal plants and 31 species of edible weedy plants.

A total of 55 species are used by their edible fruits and 34 produce edible leaves, including 27 species of the traditional greens called “quilitl” (“quilite” in plural) in Náhuatl whose young tender leaves are consumed and are among the most important food resources in rural communities of the study area. Other 19 species are used for their leaves to wrap food during its preparation which are condiments conferring flavor to food. Additionally, we found that seeds of 15 species are edible, the whole plant of 10 species are harvested; and rhizomes of 7 species, inflorescences of 6 species, flowers of four species, stems of four species and tendrils of two species are also consumed as food.

A total of 61 species are marketed at both local and/or regional levels thus providing significant monetary incomes to households. From the commercialized species, 48 are seasonally sold when the resources are available, wild species in this condition have some management type. A total of 13 plant species commercialized are available throughout the whole year, most of them being cultivated-domesticated plants and some others wild, weedy and ruderal plants.

### Management types

Most edible plant species are obtained through simple gathering but 33 species had additionally other type of management (Table 1). From these species, local people profit leaves, flowers, fruits and/or whole plants. Most species have more than one useful part, but we identified those used: 1) mostly by their vegetative parts (16 species, 48.5%); 2) mostly by their reproductive parts (11 species, 33.3%) including flowers, fruits, flower buds, inflorescences and seeds; and 3) mostly complete individuals (6 species, 18.2%) (Table 1).

We recorded 11 species that are tolerated in maize fields (milpas) and homegardens. These are species with a) low frequencies in natural vegetation due to massive extraction associated to their increasing economic importance in recent years; or b) declining cultural importance and for which tolerance is progressively unusual (see Additional file 2). The following forms of deliberate propagation were identified among managed plant species: 1) mainly by seeds (11 species, 33.3%); 2) mainly by vegetative parts (5 species, 15.1%); 3) mainly by transplanting entire individual plants (8 species, 24.2%); and 4) propagation through several forms (9 species, 27.3%), including seeds, vegetative propagules and transplanting of complete individuals from forests to homegardens and crop fields. Plant species that in the last 20 years have increased their economic value were found under especial effort of deliberate propagation. Several species reported not to be intentionally propagated were reported by people to receive management practices such as pruning, soil removal, elimination of competitors, which may increase availability of their products.

Seven species (21%) are clearly managed by women. These are species growing in homegardens and others spaces near peoples’ homes. Harvest of edible weeds (“quilite”) is considered a female activity. For instance, *Cleoserrata speciosa* should be properly harvested by experienced women since, depending on the way in which the leaves are cut, "quilite" acquire particular flavor. If harvesting of leaves is not carefully performed (cutting the leaves with nails, just below the petiole), cooked “quilite” will be bitter. Preparation of this species is a slow and complicated process (it takes a whole day, requiring continually adding water and firewood to keep constant cooking temperature, ash for acquiring the desired color, and a bit of salt until “quilite” are completely cooked), that requires experience that only have the best cooks.

Similarly, management of some species is considered typically practiced by men. These are those needing climbing trees, handling thorny plants or entering into inaccessible places (ravines, cliffs, among others) for harvesting edible parts. For instance, harvesting sap of Agave salmiana used to produce the fermented beverage called "pulque” requires knowledge and tools that local culture has assigned to men. Specifically, the "tlachiqueros", people who are dedicated to extract agave sap and preparing “pulque” is considered a guild of men. Other examples are Eugenia capuli and Quercus candicans, trees more than 20 m tall whose fruit and leaves harvesting requires men to climb up.

### Artificial selection criteria

A crucial aspect of artificial selection is the recognition of variants in populations, some of them having favorable characteristics to humans, the continuous selection favoring particular variants leading (at least in theory) to domestication. We found that in 21 of the 33 native managed species (63.3%), the interviewees explicitly recognized particular variants preferred for using, and all of them are species managed through deliberate propagation (Table 7). For instance, four variants of *Chamaedorea tepejilote* are recognized, people preferring those producing greater biomass inflorescences which can be commercialized at higher prices. Similarly, for *Dasylirion serratifolium* people recognize two varieties, although collecting both varieties involves similar effort people direct their efforts to collect the most productive variant which has higher price on the market. For *Porophyllum ruderale* people identify two variants, “white” and “purple”, the white one being widely cultivated in warm humid areas whereas the purple variety occurs in areas with dry and warm climate. The latter variety is less consumed and not cultivated. In other species, variation is more subtle and unclear the preference in favor of one particular variant.

Regional market preferences are influencing patterns of selection. For instance, in “tempesquistle” *Sideroxylon palmeri* round fruits are preferred over ellipsoid fruits. Consumers of the lowlands of the Tehuacán Valley consider that ellipsoid fruits have higher content of latex and for this reason this variety is considered wild ("tempesquistle de monte"). *Litsea glaucescens* has two varieties, one producing dark green leaves with light odor, the other with bcenter green leaves and stronger odor. Although both are harvested and commercialized, consumers prefer the variant of bcenter green leaves and strong odor. Selection criteria that are important at regional level are not necessarily consistent with those predominating at local level.

### Management intensity

Gathering may be conducted with different intensity among plant species. It is possible to distinguish two forms of harvesting edible plant species: a) dedicated harvest; which is a collection planned or programmed, and b) opportunistic harvest which is a not planned harvest, performed sporadically while doing other activities. Dedicated harvest was recorded in 18 species (54% of total) whereas opportunistic harvest was recorded in 6 species (18%). For 9 species (27%) the form of harvest was unclear since interviewees reported contradictory information (Additional file 2).

A total of 15 species (45%) are perceived by local people as easy to harvest since they can be found close to their homes, there is no need to use tools to harvest or processing, and are easily manipulated. Most edible weeds or “quilite”, among them *Phytolacca icosandra*, *Piper auritum and Solanum americanum* are considered easy to harvest. On the contrary, 8 species (24%) are considered difficult to harvest and require special techniques. This is the case of *Agave salmiana* whose management involves specialized knowledge and special tools (metal scrapers, dibble, and “acocote” *Lagenaria siceraria*, used to suck the sap from agaves stems). Another example is *Yucca elephantipes*, whose inflorescences harvesting requires people to climb up between sharp and pointed leaves to heights up to 5 m. In other cases difficulties of harvesting are associated with the time devoted for searching and extraction, as well as the distance from people’s homes to plants’ populations. For instance, walking half a day to get a resource is considered a heavy and difficult activity. These are the cases of *Peperomia peltillimba* and *Spathiphyllum cochlearispathum* (Additional file 2). No tools are involved in management of 21 edible plant species (64%), whereas 12 species (36%) require using tools often knives, machetes, picks and mattock (Additional file 2).

According to Table 8, the lowest values of management intensity correspond to plant species under simple gathering or tolerance. Most of them are annual abundant plants, consumed occasionally by few people. These are the cases of *Phytolacca icosandra, Vaccinium leucanthum*, *Tigridia pavonia*, and Sonchus oleraceus. However, some species with low management intensity values have restricted distribution these are the cases of *Sideroxylon palmeri* and *Cleoserrata speciosa*. In contrast, plant species having high management intensities are those with economic importance, mostly perennial species with recognized variants and several propagation forms whose management requires using tools, that are protected by collective regulations (Table 8).

**Table 8:** Management intensity and risk indexes calculated per edible plant species studied base don th scores of the first principal component of PCA analyses

**Species**	**Management intensity index**	**Risk index**
*Amaranthus hybridus*	0.10253	0.50874
*Agave obscura*	0.00051	-0.5632
*Agave salmiana*	1.54163	2.80585
*Brassica rapa*	-0.37473	-0.39934
*Chamaedorea tepejilote*	2.84038	2.00167
*Canna indica*	0.45666	-0.55632
*Crataegus mexicana*	0.25688	-0.43962
*Cestrum nocturnum*	0.01114	0.06298
*Cleoserrata speciosa*	-1.3656	-0.48555
*Dasylirion serratifolium*	0.91661	0.57903
*Eugenia capuli*	0.92784	0.58267
*Inga vera*	-0.14487	-1.06197
*Jatropha curcas*	-0.71903	-0.92313
*Litsea glaucescens*	1.53319	2.45691
*Leucaena leucocephala*	0.89194	0.17581
*Palismatifolia*	-0.59795	-0.44745
*Piper auritum*	0.22645	-0.49444
*Phaseolus coccineus*	0.13201	-0.04537
*Phytolaca icosandra*	-1.19325	-0.09294
*Peperomia peltilimba*	-0.18274	0.89908
*Porophyllum ruderale*	0.19413	0.88521
*Prunus serotina*	0.7972	-0.6179
*Quercus candicans*	1.63139	-0.25941
*Renealmia alpinia*	0.00994	-0.28281
*Raphanus raphanistrum*	-1.48358	-0.18893
*Solanum americanum*	-0.81386	-0.56855
*Spathiphyllum cochlearispathum*	-0.60716	-0.61467
*Sambucus mexicana*	-0.73268	-0.71948
*Sonchus oleraceus*	-1.34859	-0.41756
*Sideroxylon palmeri*	-0.94697	-0.47684
*Tigridia pavonia*	-1.30733	-0.41779
*Vaccinum leucanthum*	-0.68357	-0.68177
*Yucca elephantipes*	0.03149	-0.20291

### Spatial and temporal availability of plant resources

Most edible plant resources have a marked seasonality (70%); these are the cases of annual plant species tolerated in crop fields as well as reproductive parts of perennial species. Species that are available the whole year (30%) are perennial plants with edible vegetative parts (leaves, stems, rhizomes, etc., Table 1).

As it is shown in Table 3, *Quercus candicans* is the species perceived by people to be the most abundant, whereas *Crataegus mexicana* is the scarcest. Perception is often biased by sufficiency of the availability of a resource but not necessarily an ecological abundance in environmental units where the species is distributed. However, perception of abundance is apparently closely related to the distribution of plant species. For instance, *Eugenia capuli* is a scarce species harvested in tropical forests, which are distant from the villages included in this study; consequently, only a few individual trees found in coffee plantations and homegardens were reportedly used. Another example is *Spathiphyllum cochlearispathum*, a species with a spread growth pattern making it necessary to walk long distances to harvest its products, which apparently influences the perception that this species is scarce.

People perceive that availability of 7 species (21%) has declined, which is explained by the following reasons: a) some species have been replaced by others and therefore are not propagated with the same intensity, b) cultural changes have resulted in a decrease in consumption frequency, c) overexploitation due to increasing demand of products in regional markets. An example of the first situation is *Vaccinium leucanthum*, which in the past was used to prepare fermented and boiled ("*atole*") beverages. Currently fermented beverages are made with apple, quince and plum, all introduced species. The traditional beverage "*atole*" is now prepared with artificially flavored industrialized flour of corn and rice. Examples of the second situation are *Agave salmiana* whose decreasing availability is caused by rapid cultural changes since "*pulque*" consumption has recently been replaced by beer. Similarly, consumption of *Phaseolus coccineus* has declined since it is now considered of low cultural prestige. Examples of the third situation are *Litsea glaucescens* and *Peperomia peltillimba* whose populations have decreased due to their increasing demand in regional markets. People report that an indicator of scarcity of these resources is that now they have to go further away and take longer time to harvest them. These species have been locally used and exclusively exchanged for other products, and their populations remained relatively stable. But the opening of new roads and access to regional markets represented for local people an opportunity to obtain monetary incomes. Nowadays, regional markets demand these products in high quantities. Some people have tried to propagate these species without success.

In contrast, three species are perceived to have increased their abundance in association to their increase of commercial value. Demand in markets has enhanced people to cultivate them now but previously were only harvested in the wild. These are the cases of *Chamaedorea tepejilote*, *Renealmia alpinia*, and *Porophyllum ruderale*, which are easily propagated. For 23 species (70%) no changes in their availability were perceived by people.

People generally considered that incidence of pests is low or inexistent in the 33 native managed plant species studied. However, they mentioned that some pests attack eleven species (33%) and affect their availability in different degrees (Additional file 2). The main pests mentioned are aphids, white scale, caterpillars, moths, grasshoppers, and fungi. Although these pests damage leaves, stems and fruit, preventive or correction actions are only occasional and limited to manual removal, pruning of affected areas, and application of soapy water or lime to remove the pest. People seldom use agrochemicals for controlling pests of species tolerated in crop fields (*Amaranthus hybridus*, *Cleoserrata speciosa*, and *Phaseolus coccineus*), and when applied is a consequence of protecting the staple crops.

### Cultural and economic importance

A total of 21 managed plant species (63.6%) had an economic value, 14 of which (42.4%) are exchanged and sold, 4 (12.1%) only purchased by cash and 3 (9.0%) are only exchanged by other products (barter). In contrast, 12 species (36.3%) are not considered economically important, since their consumption is limited to a marked season or sporadic events. Moreover, for five species (15.0%) data are not conclusive, since 20% of interviewees indicated that a resource is sold, while others say it is only for household consumption (Table 3).

The highest values of cultural importance were identified in species widely and frequently consumed, with several uses, commercialized in markets and are consumed in several communities even when they are absent in their territories. These are the cases of *Chamaedorea tepejilote*, *Agave salmiana* and *Litsea glaucescens*. The lowest values of cultural importance were recorded in plant species only occasionally consumed, few use types, easily substituted by other resources and not commercialized. These are the cases of *Tigridia pavonia*, *Raphanus raphanistrum* and *Sonchus oleraceus*. *Cleoserrata speciosa* and *Sideroxylon palmeri* are economically important and appreciated as edible resources; however, their frequency of consumption is low and restricted to areas where these species grow.

### Collective regulations for accessing to edible plant resources

Collective regulations to access plant resources were documented to occur in three species (*Dasylirion serratifolium*, *Litsea glaucescens*, and *Quercus candicans*). These regulations are rules agreed in the General Assembly, which is the meeting of all household’ heads in a community, and are designed to protect resources considered important for the whole community. They include partial ban to cut these species and sanctions to those who do it. Regulations are generally respected by people but penalties range from a verbal reprimand to monetary fines. Communitarian rules protect lands and resources communally owned. Constructing collective regulations is associated with the perception that resources are being depleted or that slow-growing species have to be left growing before taking advantage of them. For instance *Litsea glaucescens* is a scarce species, and communitarian regulations prohibit gathering its leaves for commercial purposes (which frequently involves cutting the whole tree for making harvesting easier). People who violate the rule are fined $45 US dollars. People are authorized harvesting leaves of this species only once per year, the community designating specific persons (called "*mozos*"), which are the only one authorized to harvest leaves, branches or sometimes trees.

### Risk and management intensity

**Figure 3:**
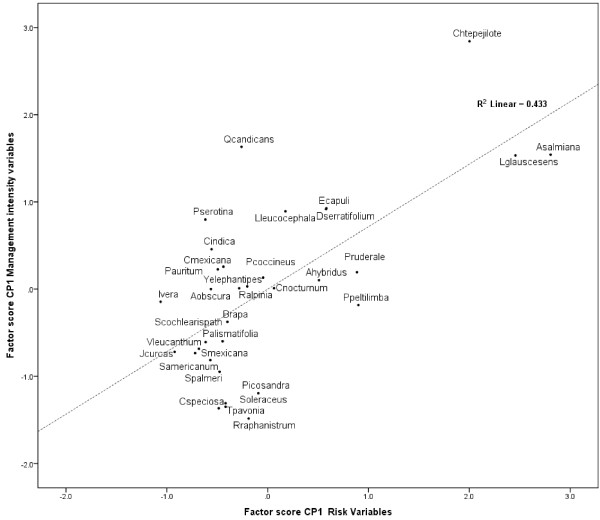
**Regression analysis of the management intensity index as a function of the risk index calculated as the scores of the first principal component of PCA analyzing indicators of these aspects per edible plant species (R**^**2 **^**= 0**.**433, P<0**.**001).**

**Figure 4:**
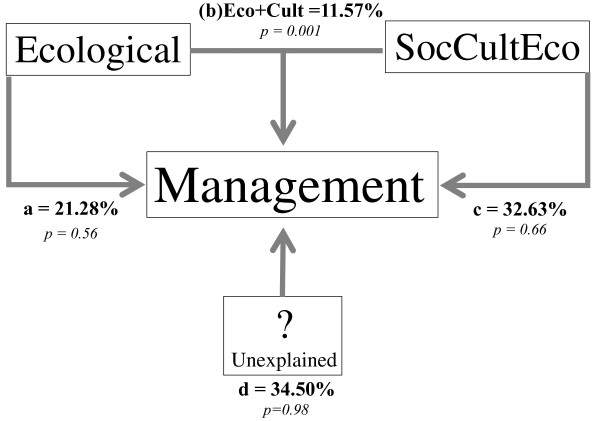
Partitioned CCA scheme showing the relative influence of ecological, sociocultural (SocCultEco) factors and their interaction on management strategies of edible plant species by Náhuatl communities of the Tehuacán Valley.

Regression analysis in Figure 3 indicates the highly significant linear relation between risk and management intensity indexes (R2 = 0.433, P<0.001). Partitioning CCA explains 65.5% of the management variation as shown in Figure 4. This variation can be explained mainly by sociocultural factors (32.6%) while ecological data explain 21.3%. Intersection of ecological and sociocultural factors explains 11.6% and is statistically significant. Unexplained variation was 34.5%. Two variables of the intersection of ecological and sociocultural indicators were particularly important: distribution and abundance of resources and number of uses (Table 9). Other variables such as number of persons commercializing and consuming plant resources were important although statistically no significant.

**Table 9:** Permutation test for CCA variables under reduced model of management factors and ecologic and sociocultural and economic factors

**Variable**	**DF**	**Chisq**	**F**	**Pr(>F)**
Quantity Marketed (kg or L)	1	0.0087	1.2515	0.46
Cost (Kg or L)	1	0.0035	0.5081	0.9
People who Sell	1	0.0189	2.7245	0.07
Number People Consume	1	0.0192	2.7721	0.1
Frequency Use	1	0.0084	1.2105	0.33
Last Day Consumption	1	0.0076	1.0914	0.43
Number of Uses	1	0.0312	4.489	0.03
Useful Parts	1	0.0114	1.6426	0.23
Number Useful Parts	1	0.0005	0.0758	1
Commercialization	1	0.0099	1.4333	0.3
Medicinal Use	1	0.0051	0.7391	0.57
Espacial Distribution	1	0.0237	3.4077	0.02
Temporal Distribution	1	0.0046	0.6602	0.67
Lyfe Cycle	1	0.0084	1.2126	0.46
Ecological Dominance	1	0.0062	0.8891	0.64
Disponibility	1	0.0017	0.2475	0.98
Reproductive System	1	0.0044	0.6357	0.72
Relative Importance Value	1	0.011	1.5811	0.22
Residual	14	0.0972		

Partitioned CCA indicates that variation of management intensity is 67.6% explained by risk variables. According to Table 10 the following variables were statistically significant: life cycle (annual or perennial), reproductive system (self-compatible or self-incompatible), distribution (broad or restricted), number of parts used, number of forms in which a resource is managed and regulations for using resources.

**Table 10:** Permutation test for CCA variables under reduced model of management intensity factors and risk factors

**Variable**	**DF**	**Chisq**	**F**	**Pr(>F)**
**Lyfe cycle**	1	0.042	10.120	0.01
**Reproductive system**	1	0.016	3.767	0.01
**Distribution**	1	0.011	2.609	0.01
Abundance perceived	1	0.006	1.474	0.30
Useful part	1	0.007	1.566	0.16
Temporal disponibility	1	0.007	1.547	0.30
Pests	1	0.004	1.059	0.46
**No. of useful parts**	1	0.014	3.235	0.01
**No. of management Forms**	1	0.012	2.855	0.02
**Rules**	1	0.030	7.213	0.01
Cultural importance	1	0.010	2.263	0.06
Economic importance	1	0.007	1.778	0.07
Spatial availability	1	0.009	0.215	0.97
Residual	19	0.079		

Variables in bold were statistically significant.

**Figure 5:**
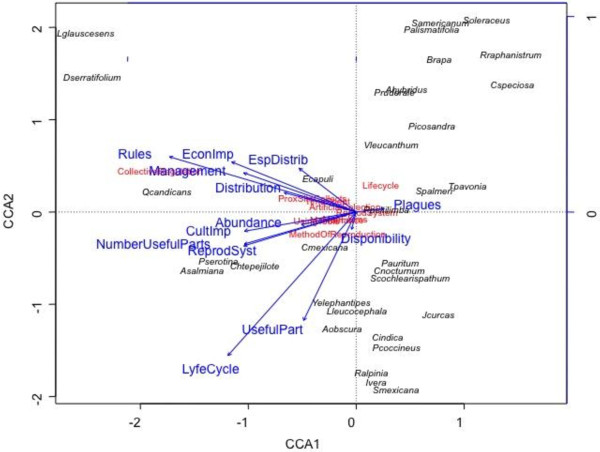
Ordination plane of CCA showing how species (italics) and management intensity (red) are influenced by risk variables (blue arrows) of edible plant species by Náhuatl communities of the Tehuacán Valley.

Figure 5 shows that long-life span species from which entire plants or their reproductive parts are used, with no regulations, high economic and cultural value and self-incompatible breeding system have higher risk. These are the cases of *Agave salmiana*, *Dasylirion serratifolium* and *Litsea glaucescens*. On the opposite end of a risk gradient, annual plant species whose vegetative parts are used and have self-compatible breeding systems have low risk levels. These are represented by weedy species in maize fields such as *Plantago alismatifolia*, *Raphanus raphanistrum*, *Solanum americanum* and *Sonchus oleraceus*.

## Discussion and conclusions

### Management types

Náhuatl people from Coyomeapan manage plants in different forms and with different intensities and that management of wild and weedy plants takes place parallel to agricultural management of domesticated plants. These management types and intensities are integrated to a subsistence pattern based on multi-purpose use of plant resources and ecosystems which is characteristic of indigenous peoples in Mexico [6].

We found general categories of management forms occurring in natural environments (*in situ* management of both wild and weedy plants), and others occurring in human-controlled environments, (*ex situ* management in crop fields, homegardens, and agroforestry systems). We also consider that recognition of intra-specific variants and practice of differential use and artificial selection on these variants are key criteria for classifying both *in situ* and *ex situ* management types. Consequently, the classification should include the following categories:

### In situ interactions

(1.1) Gathering without recognition of variants. Most wild species are gathered from natural vegetation without distinction of varieties of particular preference. Some are annual, other are perennial. *Sambucus mexicana*, *Tigridia pavonia* and *Vaccinium leucanthum* are some examples of this condition (see Additional file 2).

(1.2) Specialized gathering with appreciation of variants. The gathering is differential, since some individuals are preferred by collectors due to specific features (flavor, color, texture, size, presence or absence of toxic substances, etc.). For instance, *Quercus candicans* whose leaves are used to cook “*tamales*” on celebrations days. Collectors recognized two variants: I) “*Lisa*”. Leaves large, thin, smooth. Leaf underside glabrous and bcenter. The surface of the leaf is light green; and II) “*cucharuda*”. Leaves thick, leathery, spoon-shaped. Undersides densely tomentose, beam darker color. The “*lisa*” variant is preferred to wrap tamales, since it can be handled better and confers a nicer flavor. According to interviewees, the variety "*cucharuda*" is difficult to bend and confers bitter taste to “*tamales*”.

(2.1) Tolerance without recognition of variants. These are species tolerated in maize fields, which are tolerated considering only their availability but not variant types. People practice weeding twice per agricultural cycle and decide to maintain these plant species because they are edible. An example of these plants is *Cleoserrata speciosa*. Among perennial species *Sambucus mexicana* tolerated in limits of parcels can be mentioned.

(2.2) Tolerance of recognized variants. Some variants are preferred and deliberately tolerated when peasants open crop fields to sow maize seeds; or when this are cleaned of weeds. This is the case of annual species of "*quilite*". Many of these species behave as weeds and invade the crop fields. However, people distinguished variations in individuals, and these differences allow a differential removal of phenotypes that are undesirable. For instance, *Raphanus raphanistrum* is a weedy species of which two varieties are recognized: I) Pubescent leaf, purple flower, intense flavor and hard to digest; and II) Smooth leaf, white flower and sweeter taste. This latter variety is occasionally used as food. The pubescent variety is eliminated, since its consumption can cause stomach ache, or alternatively is used as fodder for turkeys, chickens, sheep, goats, and pigs. The same is true for *Phytolacca icosandra*. Another example of tolerance, but for a perennial species, is *Dasylirion serratifolium*, for which two varieties are recognized: I) Individuals with purple inflorescence and larger flower buds; and II) Individuals with white inflorescence and smaller flower buds. Both varieties are consumed and traded, but if a person needs to make a choice she/he will selectively remove the variety with white inflorescences, since it produces a lower yield and has lower market prices.

(3.1) Enhancement without recognition of variants. This management type includes practices directed to deliberately increase abundance of a plant species but not specific variants. This is the case of *Phaseolus coccineus*, whose seeds are manually dispersed in parcels where it is absent. Once in the parcel, people do not report investing any additional action.

(3.2) Enhancement with recognition of variants. In this management type different strategies are undertaken to increase the population density of useful plants. This includes the sowing of seeds or the intentional propagation of vegetative structures in the same places occupied by populations of wild or weedy plants. For instance, *Brassica rapa*; and *Solanum americanum* are enhanced in the crop fields. In addition, the seeds of *Phytolacca icosandra*, *Porophyllum ruderale*, and *Sonchus oleraceus* are scattered on roads and crop fields in fallow. An example of perennial plants managed in this form is the scattering of seeds and vegetative propagules of *Agave obscura* in areas around mother plants.

(4.1) Protection without recognition of variants. This management practice includes actions directed to preserve wild plant resources that are not cultivated nor transplanted. In this type of action we include those practiced without distinction of variants. This can be reported for *Agave obscura* in which all plants recognized as competitors are removed. Also, dry leaves are removed in order to favour production of edible flower buds and it is a protection action.

(4.2) Protection with recognition of variants. It consists of actions that seek to preserve wild resources without being cultivated or transplanted. These actions may be practical, as in the case of *Litsea glaucescens*, whose members are sometimes surrounded with branches to prevent grazing of goats and sheep. Another example are regulations intended to safeguard scarce species such as *Agave salmiana*, whose leaves are used to prepare “*barbacoa*” (earth oven cooked meat).

### *Ex situ* interactions

Some wild plants are cultivated in homegardens or in edges of crop fields. They are propagated by seeds, vegetative propagules, and in most cases by transplanting whole plants. The *ex situ* interactions may be through:

Seeds sowing. As in the case of *Chamaedorea tepejilote*, whose fruits are edible and the seeds are spread in homegardens, coffee plantations, and occasionally in cornfields. Other examples are *Amaranthus hybridus*, *Canna indica*, *Prunus serotina*, *Jatropha curcas*, *Renealmia alpinia*, *Leucaena leucocephala*, *Porophyllum ruderale*, *Sideroxylon palmeri* and *Cleoserrata speciosa* (see Additional file 2).

Transplanting of whole plants. This practice involves transplanted complete individuals from wild environments to crop field or homegardens. Frequently, these individuals have characteristics that are appreciated by people. For instance, *Agave obscura* and *Eugenia capuli* are transplanted to homegardens because according to people these are scarce in natural vegetation. Their proximity allows them to take advantage of these species without traveling long distances. *Eugenia capuli* has recently acquired importance as flavoring for coffee, so this is an additional reason for transplanting it. *Crataegus mexicana* is also transplanted to crop fields. Besides eating the fruit, the whole plant it is used to graft of fruit trees like apple and quince. Other species transplanted are *Inga vera*, *Peperomia peltilimba* and *Spathiphyllum cochlearispathum*. *Agave salmiana* is propagated mainly in crop fields. Shoots are removed of mother plants and they are replanted mainly on the edges of crop fields. These actions allow the conservation of soil and retain moisture. Also they serve as a living fences to demarcate plots.

Propagation of vegetative parts. Stems of *Cestrum nocturnum* and *Piper auritum* whose leaf buds are edible, are planted in homegardens.

### Management intensity

It is possible to generally recognize a gradient of management intensity. How intense plant management can be appears to be related mainly with the economic and cultural importance, easiness of propagation, as well as perception of resource scarcity (Table 8, Figure 3).

With the exception of *Porophyllum ruderale* and *Amaranthus hybridus*, most of managed plant species recorded in this study are perennial plants, particularly those with both sexual and asexual propagation. This is for instance the case of *Chamaedorea tepejilote*. In contrast, most species with low levels of management intensity are those propagated by either sexual or asexual means.

Economic importance enhances plant management intensity. Species like *Chamaedorea tepejilote*, *Leucaena leucocephala*, *Litsea glaucescens*, *Eugenia capuli* and *Porophyllum ruderale* are now demanded in regional markets and people are interested in ensuring and increasing their availability through propagating them.

We found that the management intensity index proposed is generally proportional to the risk index constructed based on distribution, abundance, cultural and economic importance. This general pattern suggests that in the case of edible plants, management is a response associated to food security. Uncertainty in the availability of edible resources appears to be an important motive of management. Such uncertainty should be perceived associated to ecological factors such as year to year periods according to variations in mean temperature and precipitation, longer period’s climate change, pests’ incidence, among others. Meaning of uncertainty or scarcity may be variable according to variation in cultural and economic importance. How uncertainty or scarcity of a plant resource is meaningful to people may be influenced by the role of plants in human subsistence and how indispensable or substitutable the resources are. Uncertainty in plant availability is an important issue to be investigated in order to understand motives of plant management and domestication. Uncertainty could also be the a motive of management of plants used for medicinal purposes or for fuel wood whose availability people want to ensure, to have closer to their houses or to increase. Ornamental plants are importantly managed and uncertainty could not be the main factor motivating management since these plants are managed because of their beauty and the spiritual wellbeing they determine when having them around. Therefore, motives of plant management would not only be responses to scarcity and food security, but this is a topic yet to be investigated. Qualitative research approaches would be appropriate for a deeper understanding on this topic.

In a previous study Casas et al. [13,19] discussed that intensity of interactions between humans and plants are influenced by 1) their role in human subsistence (in economic and cultural terms), 2) their availability (distribution and abundance) in relation to human demand, 3) quality of their products, 4) viability of managing their propagules, their populations or biotic communities where these resources occur, which is influenced by length of life cycle, reproductive system, capacity of adaptation to human made environments, among others. Our current study confirms that these are relevant aspects and provides methodological tools for analyzing how meaningful these factors are for motivating management. These factors are dynamic throughout time and those motivating management at present are most probably different to those motivating management in the past. However, understanding principles of management construction is helpful for analyzing how humans currently make and made decisions in the past, as well as for designing decisions for a sustainable future.

## Competing interests

The authors articulate that they have no competing interest.

## Authors' contributions

JB main author, involved in the study design, conducting of interview, field work, literature review and general data collection and systematization, wrote the first draft and concluded the final version this paper. AC main coordinator-supervisor of the research project; contributed with original data and the designing of all the researches providing the information for the current analysis; participated in fieldwork, systematization and analysis of data and reviewed several drafts of the manuscript. EV suggested and performed statistical analysis, teaming with JB and AC. DP and JC contributed to designing and following progress of the research and field work and data analyses. All authors read and approved the final manuscript.

## Authors' information

JB postgraduate student at the Centro de Investigaciones en Ecosistemas (CIEco), UNAM. AC, DP and EV full time researchers at CIEco, UNAM. JC researcher at the Jardín Botánico, Instituto de Biología, UNAM.

## Supplementary Material

Additional file 1 **Edible plant species recorded in the study area.** Voucher specimens are José Blancas collection numbers. Click here for file

Additional file 2 Management forms of edible plant species in Santa María Coyomeapan, México. Click here for file
