# Supplementary material for: Ecological and socio-cultural factors influencing plant management in Náhuatl communities of the Tehuacán Valley, Mexico
Source: J Ethnobiol Ethnomed. 2013 Jun 2;9:39. doi: 10.1186/1746-4269-9-39 (PMC3702518; doi:10.1186/1746-4269-9-39)
Supplement: Additional file 1 — Edible plant species recorded in the study area. Voucher specimens are José Blancas collection numbers. [file 1746-4269-9-39-S1.docx]

**Appendix 1.** Edible plant species recorded in the study area. Voucher specimens are José Blancas collection numbers.

| ***Species*** | ***Common name*** | ***Ecological status*** | ***Origin status*** | ***Reproduction Cycle*** | ***Lifestyle*** | ***Useful parts*** | ***E. I.*** | ***Disponibiliy*** | ***Voucher number*** |
| --- | --- | --- | --- | --- | --- | --- | --- | --- | --- |
| **Adoxaceae** |  |  |  |  |  |  |  |  |  |
| *Sambucus mexicana* C. Presl ex DC. | Xometl | Wild | Native | Perennial | Shrub | Leaves |  | Continual | 2088, 2218, 2223 |
| **Agavaceae** |  |  |  |  |  |  |  |  |  |
| *Agave obscura* Schiede | Cacaya | Wild | Native | Perennial | Rosette | Inflorescence |  | Seasonally | 2003, 2211 |
| *Agave americana* L. | Flor de Maguey | Domesticated | Native | Perennial | Rosette | Stem |  | Seasonally | 2001 |
| *Agave salmiana* Otto ex Salm-Dyck | Maguey de pulque | Domesticated | Native | Perennial | Rosette | Stem | * | Seasonally | 2002 |
| *Yucca elephantipes* Regel | Izote | Domesticated | Native | Perennial | Rosette | Inflorescence |  | Seasonally | 2104, 2185 |
| **Amaranthaceae** |  |  |  |  |  |  |  |  |  |
| *Amaranthus hybridus* L. | Baquilitl | Ruderal, Weedy | Native | Annual | Herb | Leaves |  | Seasonally | 2004, 2191 |
| **Anacardiaceae** |  |  |  |  |  |  |  |  |  |
| *Cyrtocarpa procera* Kunth | Chupandilla | Wild | Native | Perennial | Tree | Fruit |  | Seasonally | 2031 |
| *Mangifera indica* L. | Mango | Domesticated | Exotic | Perennial | Tree | Fruit |  | Seasonally | 2051 |
| **Annonaceae** |  |  |  |  |  |  |  |  |  |
| *Annona cherimola* Mill. | Chirimoya | Domesticated | Exotic | Perennial | Tree | Fruit | * | Seasonally | 2005, 2170 |
| *Annona muricata* L. | Guanábana | Wild | Native | Perennial | Tree | Fruit | * | Seasonally | 2006 |
| **Apiaceae** |  |  |  |  |  |  |  |  |  |
| *Daucus carota* L. | Zanahoria | Domesticated | Exotic | Annual | Herb | Rizome |  | Continual | 2033 |
| *Eryngium foetidum* L. | Vitzcolantro | Ruderal, Weedy | Native | Annual | Herb | Leaves |  | Continual | 2162, 2253 |
| **Araceae** |  |  |  |  |  |  |  |  |  |
| *Spathiphyllum cochlearispathum* (Liebm.) Engl. | Elotlquilitl | Weedy | Native | Perennial | Herb | Inflorescence | * | Seasonally | 2098, 2197 |
| *Xanthosoma robustum* Schott | Bebetcho | Weedy | Native | Perennial | Herb | Rizome |  | Continual | 2103 |
| **Arecaceae** |  |  |  |  |  |  |  |  |  |
| *Acrocomia mexicana* Karw. | Coyol | Wild | Native | Perennial | Palm | Fruit | * | Seasonally | 2011 |
| *Chamaedorea tepejilote* Liebm. ex Mart | Tepexilotl | Wild | Native | Perennial | Palm | Leaves, Inflorescence bud, apical meristem | * | Seasonally | 2013, 2202, 2316 |
| **Asparagaceae** |  |  |  |  |  |  |  |  |  |
| *Dasylirion serratifolium* (Karw. ex Schult. f.) Zucc. | Mazitzi | Wild | Native | Perennial | Rosette | Inflorescence | * | Seasonally | 2032 |
| **Asteraceae** |  |  |  |  |  |  |  |  |  |
| *Gymnosperma glutinosum* (Spreng.) Less. | Popotl | Ruderal, Weedy | Native | Perennial | Herb | Whole plant |  | Continual | 2211, 2222 |
| *Lactuca sativa* L. | Lechuga | Domesticated | Exotic | Annual | Herb | Leaves |  | Continual | 2046 |
| *Porophyllum linaria* (Cav.) DC. | Pipitza | Weedy | Native | Annual | Herb | Whole plant | * | Seasonally | 2208, 2213 |
| *Porophyllum ruderale* (Jacq.) Cass. | Papaloquilitl | Weedy | Native | Annual | Herb | Leaves | * | Continual | 2072, 2216 |
| *Sonchus oleraceus* L. | Memella | Ruderal, Weedy | Exotic | Perennial | Herb | Leaves |  | Seasonally | 2097 |
| *Tagetes filifolia* Lag. | Anís | Ruderal, Weedy | Native | Annual | Herb | Whole plant |  | Continual | 2217 |
| **Begoniaceae** |  |  |  |  |  |  |  |  |  |
| *Begonia caroliniifolia* Regel | Cuajtomatl | Wild | Native | Perennial | Herb | Rizome |  | Continual | 2012 |
| **Bignoniaceae** |  |  |  |  |  |  |  |  |  |
| *Parmentiera aculeata* DC. | Cuajilote | Wild | Native | Perennial | Tree | Fruit | * | Seasonally | 2492 |
| **Bixaceae** |  |  |  |  |  |  |  |  |  |
| *Bixa orellana* L. | Axiote | Domesticated | Native | Perennial | Tree | Seed | * | Seasonally | 2007 |
| **Brassicaceae** |  |  |  |  |  |  |  |  |  |
| *Brassica juncea* L. | Mostaza | Ruderal, Weedy | Exotic | Annual | Herb | Whole plant |  | Seasonally | 2008, 2227 |
| *Brassica rapa* L. | Colesh | Ruderal, Weedy | Exotic | Annual | Herb | Whole plant |  | Seasonally | 2003, 2009, 2226 |
| *Raphanus raphanistrum* L. | Rabanoquilitl | Ruderal, Weedy | Exotic | Annual | Herb | Leaves, Stem |  | Seasonally | 2082, 2173 |
| *Rorippa nasturtium-aquaticum* (L.) Hayek | Atlanquilitl | Weedy | Exotic | Perennial | Herb | Whole plant |  | Seasonally | 2084 |
| **Cactaceae** |  |  |  |  |  |  |  |  |  |
| *Hylocereus undatus* (Haw.) Britton & Rose | Pitahaya | Domesticated | Native | Perennial | Creeper | Fruit | * | Seasonally | 2041 |
| *Opuntia ficus-indica* (L.) Mill. | Nopal | Domesticated | Native | Perennial | Shrub | Stem | * | Continual | 2057 |
| **Cannaceae** |  |  |  |  |  |  |  |  |  |
| *Canna indica* L. | Panispatl | Ruderal | Native | Perennial | Herb | Leaves |  | Continual | 2163 |
| **Capparaceae** |  |  |  |  |  |  |  |  |  |
| *Cleoserrata speciosa* (Raf.) H.H. Iltis | Mabilquilitl | Weedy | Native | Annual | Herb | Leaves | * | Seasonally | 2217 |
| **Caricaceae** |  |  |  |  |  |  |  |  |  |
| *Carica papaya* L. | Papaya | Domesticated | Native | Perennial | Tree | Fruit | * | Seasonally | 2013 |
| **Chenopodiaceae** |  |  |  |  |  |  |  |  |  |
| *Chenopodium ambrosioides* L. | Epazote | Ruderal, Weedy | Native | Annual | Herb | Whole plant |  | Continual | 2015 |
| *Chenopodium berlandieri* Moq. | Quelite blanco | Ruderal, Weedy | Native | Annual | Herb | Leaves |  | Continual | 2014 |
| *Chenopodium nuttalliae* Saff. | Huauzontle | Domesticated | Native | Annual | Herb | Inflorescence |  | Seasonally | 2010, 2016 |
| **Chrysobalanaceae** |  |  |  |  |  |  |  |  |  |
| *Couepia polyandra (Kunth) Rose* | Zapote de niño | Wild | Native | Perenne | Tree | Fruit | * | Seasonally | 2206, 2207, 2569 |
| **Convolvulaceae** |  |  |  |  |  |  |  |  |  |
| *Ipomoea batatas* (L.) Lam. | Camote | Domesticated | Native | Perennial | Herb | Rizome | * | Seasonally | 2044 |
| **Cucurbitaceae** |  |  |  |  |  |  |  |  |  |
| *Cucumis sativus* L. | Pepino | Domesticated | Native | Annual | Herb | Fruit |  | Seasonally | 2024 |
| *Cucurbita ficifolia* L. | Chilacayote | Domesticated | Native | Annual | Herb | Fruit, seeds |  | Seasonally | 2025 |
| *Cucurbita moschata* Duchesne | Abayotli | Domesticated | Native | Annual | Herb | Fruit, seeds |  | Seasonally | 2027 |
| *Cucurbita pepo* L. | Calabaza | Domesticated | Native | Annual | Herb | Fruit, seeds |  | Seasonally | 2028 |
| *Sechium edule* (Jacq.) Sw. | Chayote | Domesticated | Native | Annual | Herb | Fruit, Tendrils | * | Continual | 2089 |
| *Sicana odorifera* (Vell.) Naudin | Calabaza melón | Domesticated | Exotic | Annual | Herb | Fruit |  | Seasonally | 2000 |
| *Sicyos parviflorus* Willd. | Camachichio | Ruderal, Weedy | Native | Annual | Herb | Tendrils |  | Seasonally | 2090 |
| **Ebenaceae** |  |  |  |  |  |  |  |  |  |
| *Diospyros digyna* Jacq. | Tliltlzapotl | Wild | Native | Perennial | Tree | Fruit | * | Seasonally | 2034, 2204 |
| **Ericaceae** |  |  |  |  |  |  |  |  |  |
| *Vaccinium leucanthum* Schltdl. | Tetzmolli | Wild | Native | Perennial | Tree | Fruit |  | Seasonally | 2101, 2181, 2434 |
| **Euphorbiaceae** |  |  |  |  |  |  |  |  |  |
| *Jatropha curcas* L. | Piñón | Wild | Native | Perennial | Shrub | Seed | * | Seasonally | 2043 |
| **Fabaceae** |  |  |  |  |  |  |  |  |  |
| *Erythrina americana* Mill. | Iquimite | Domesticated | Native | Perennial | Tree | Flower |  | Seasonally | 2036 |
| *Inga vera* Willd. | Topetli | Wild | Native | Perennial | Tree | Fruit |  | Seasonally | 2437 |
| *Leucaena esculenta* (Moc. & Sessé ex DC.) Benth. | Guaje | Wild | Native | Perennial | Tree | Seed, Leaves buds | * | Seasonally | 2047 |
| *Leucaena leucocephala* (Lam.) de Wit | Guaje blanco | Wild | Native | Perennial | Tree | Seed, Leaves buds | * | Seasonally | 2214 |
| *Phaseolus coccineus* L. | Ilamatzin | Ruderal, Weedy | Native | Perennial | Shrub | Seed, Flower, Leaves | * | Seasonally | 2205, 2220, 2063 |
| *Phaseolus vulgaris* L. | Pitza | Domesticated | Native | Annual | Herb | Seed | * | Seasonally | 2167 |
| *Pisum sativum* L. | Chícharo | Domesticated | Exotic | Annual | Herb | Seed |  | Seasonally | 2069 |
| *Vicia faba* L. | Haba | Domesticated | Exotic | Annual | Herb | Seed |  | Seasonally | 2004, 2102 |
| **Fagaceae** |  |  |  |  |  |  |  |  |  |
| *Quercus candicans* Née | Tamashibitl | Wild | Native | Perennial | Tree | Leaves |  | Continual | 2210, 2219 |
| **Iridaceae** |  |  |  |  |  |  |  |  |  |
| *Tigridia pavonia* (L. f.) DC. | Tlalteztli | Ruderal, Weedy | Native | Perennial | Herb | Rizome |  | Continual | 2100 |
| **Juglandaceae** |  |  |  |  |  |  |  |  |  |
| *Juglans regia* L. | Nuez | Domesticated | Exotic | Perennial | Tree | Seed |  | Seasonally | 2045 |
| **Lamiaceae** |  |  |  |  |  |  |  |  |  |
| *Clinopodium mexicanum* (Benth.) Govaerts | Payaniltzin | Wild | Native | Annual | Shrub | Whole plant |  | Continual | 2193, 2234 |
| *Mentha piperita* L. | Hierbabuena | Domesticated | Exotic | Perennial | Herb | Leaves |  | Continual | 2005 |
| **Lauraceae** |  |  |  |  |  |  |  |  |  |
| *Litsea glauscesens* Kunth | Laurel | Wild | Native | Perennial | Tree | Leaves | * | Continual | 2050, 2190, 2208, 2439 |
| *Persea americana* Mill. | Aguacate | Domesticated | Native | Perennial | Tree | Fruit, Leaves | * | Seasonally | 2062, 2224 |
| *Persea schiedeana* Nees | Chinene | Wild | Native | Perennial | Tree | Fruit |  | Seasonally | 2017 |
| **Moraceae** |  |  |  |  |  |  |  |  |  |
| *Ficus carica* L. | Higo | Domesticated | Exotic | Perennial | Tree | Fruit |  | Seasonally | 2039 |
| **Musaceae** |  |  |  |  |  |  |  |  |  |
| *Musa x paradisiaca* L. | Xochicualli | Domesticated | Exotic | Perennial | Herb | Fruit | * | Continual | 2056 |
| **Myrtaceae** |  |  |  |  |  |  |  |  |  |
| *Eugenia capuli* (Schltdl. & Cham.) Hook. & Arn. | Mototetl | Wild | Native | Perennial | Tree | Fruit, Flower, Leaves | * | Seasonally | 2038, 2199 |
| *Psidium guajava* L. | Guayaba | Domesticated | Native | Perennial | Tree | Fruit | * | Seasonally | 2078 |
| **Oxalidaceae** |  |  |  |  |  |  |  |  |  |
| *Oxalis corniculata* L. | Limonadas | Weedy | Native | Annual | Herb | Leaves |  | Continual | 2058 |
| **Passifloraceae** |  |  |  |  |  |  |  |  |  |
| *Passiflora edulis* Sims | Maracuyá | Domesticated | Exotic | Perennial | Creeper | Fruit | * | Seasonally | 2060 |
| *Passiflora ligularis* Juss. | Granadilla | Domesticated | Exotic | Perennial | Creeper | Fruit |  | Seasonally | 2059 |
| **Phytolaccaceae** |  |  |  |  |  |  |  |  |  |
| *Phytolacca icosandra* L. | Molquilitl | Ruderal, Weedy | Native | Annual | Herb | Leaves |  | Continual | 2066, 2179, 2237 |
| **Piperaceae** |  |  |  |  |  |  |  |  |  |
| *Peperomia maculosa* (L.) Hook. | Tehuantequilitl | Wild | Native | Perennial | Herb | Leaves, Stem |  | Continual | 2108, 2199 |
| *Peperomia peltilimba* C. DC. | Tequelite | Wild | Native | Perennial | Herb | Leaves, Stem | * | Continual | 2061, 2201 |
| Piper auritum Kunth | Tlanilpayilit | Ruderal, Weedy | Native | Perennial | Shrub | Leaves |  | Continual | 2068, 2236, 2438 |
| **Plantaginaceae** |  |  |  |  |  |  |  |  |  |
| *Plantago* alismatifolia Pilg. | Toro Lengua | Ruderal, Weedy | Exotic | Annual | Herb | Leaves |  | Seasonally | 2066, 2179, 2237 |
| **Platanaceae** |  |  |  |  |  |  |  |  |  |
| *Platanus mexicana* Moric. | Papaloguitl | Wild | Native | Perennial | Tree | Leaves |  | Continual | 2070, 2230, 2442, 2471 |
| **Poaceae** |  |  |  |  |  |  |  |  |  |
| *Cymbopogon citratus* (DC.) Stapf | Zacate Limón | Weedy | Exotic | Perennial | Herb | Leaves | * | Continual | 2030 |
| *Saccharum officinarum* L | Caña de azúcar | Domesticated | Exotic | Annual | Herb | Stem | * | Seasonally | 2087 |
| *Zea mays* L. | Maíz | Domesticated | Native | Annual | Herb | Seed, Leaves | * | Seasonally | 2105 |
| **Portulacaceae** |  |  |  |  |  |  |  |  |  |
| *Portulaca oleracea* L. | Verdolaga | Ruderal, Weedy | Native? | Annual | Herb | Whole plant | * | Continual | 2073 |
| **Punicaceae** |  |  |  |  |  |  |  |  |  |
| *Punica granatum* L. | Granada | Domesticated | Exotic | Perennial | Shrub | Fruit | * | Seasonally | 2079 |
| **Rosaceae** |  |  |  |  |  |  |  |  |  |
| *Crataegus mexicana* Moc. & Sessé ex DC. | Tejocote | Wild | Native | Perennial | Tree | Fruit |  | Seasonally | 2023, 2232, 2427 |
| *Cydonia oblonga* Mill. | Membrillo | Domesticated | Exotic | Perennial | Tree | Fruit |  | Seasonally | 2029 |
| *Eriobotrya japonica* (Thunb.) Lindl. | Níspero | Domesticated | Exotic | Perennial | Tree | Fruit | * | Seasonally | 2035 |
| *Fragaria mexicana* Schltdl. | Fresa | Domesticated | Exotic | Annual | Herb | Fruit |  | Seasonally | 2040, 2193 |
| *Malus pumila* Mill. | Manzana | Domesticated | Exotic | Perennial | Tree | Fruit | * | Seasonally | 2052 |
| *Prunus americana* Marsh. | Ciruela | Domesticated | Exotic | Perennial | Tree | Fruit | * | Seasonally | 2074 |
| *Prunus armeniaca* L. | Chabacano | Domesticated | Exotic | Perennial | Tree | Fruit | * | Seasonally | 2075 |
| *Prunus persica* (L.) Batsch | Durazno | Domesticated | Exotic | Perennial | Tree | Fruit | * | Seasonally | 2076, 2165 |
| *Prunus serotina* Ehrh. | Capulín | Wild | Native | Perennial | Tree | Fruit, Seeds | * | Seasonally | 2077, 2233 |
| *Pyrus communis* L. | Pera | Domesticated | Exotic | Perennial | Tree | Fruit | * | Seasonally | 2080, 2225 |
| *Pyrus malus* L. | Perón | Domesticated | Exotic | Perennial | Tree | Fruit | * | Seasonally | 2081 |
| *Rubus eriocarpus* Liebm. | Mora | Wild | Native | Perennial | Shrub | Fruit |  | Continual | 2085 |
| *Rubus pringlei* Rydb. | Xoxonte | Wild | Native | Perennial | Shrub | Fruit |  | Continual | 2086, 2235 |
| **Rubiaceae** |  |  |  |  |  |  |  |  |  |
| *Coffea arabica* L. | Café | Domesticated | Exotic | Annual | Shrub | Seed | * | Seasonally | 2021 |
| **Rutaceae** |  |  |  |  |  |  |  |  |  |
| *Citrus aurantifolia* (Christm.) Swingle | Lima | Domesticated | Exotic | Perennial | Tree | Fruit | * | Seasonally | 2017 |
| *Citrus aurantium* L. | Limón | Domesticated | Exotic | Perennial | Tree | Fruit | * | Seasonally | 2018 |
| *Citrus reticulata* Blanco | Mandarina | Domesticated | Exotic | Perennial | Tree | Fruit | * | Seasonally | 2019 |
| *Citrus sinensis* (L.) Osbeck | Naranja | Domesticated | Exotic | Perennial | Tree | Fruit | * | Seasonally | 2149 |
| **Sapotaceae** |  |  |  |  |  |  |  |  |  |
| *Manilkara zapota* (L.) P. Royen | Zapote blanco | Wild | Native | Perennial | Tree | Fruit | * | Seasonally | 2054 |
| *Pouteria sapota* (Jacq.) H.E. Moore & Stearn | Cuatzapotl | Wild | Native | Perennial | Tree | Fruit | * | Seasonally | 2053 |
| *Sideroxylon palmeri* (Rose) T.D. Penn. | Tempesquistle | Wild | Native | Perennial | Tree | Fruit | * | Seasonally | 2091, 2476 |
| **Solanaceae** |  |  |  |  |  |  |  |  |  |
| *Capsicum annuum var. annuum* | Chiltepe | Wild | Native | Annual | Herb | Fruit |  | Seasonally | 2168 |
| *Capsicum annuum var. glabriusculum* (Dunal) Heiser & Pickersgill | Piquín | Domesticated | Native | Annual | Herb | Fruit | * | Seasonally | 2379 |
| *Capsicum pubescens* Ruiz & Pav. | Canario | Domesticated | Exotic | Annual | Herb | Fruit | * | Seasonally | 2381, 2587 |
| *Cestrum nocturnum* L. | Zopeliquilitl | Wild | Native | Perennial | Shrub | Leaves | * | Continual | 2155, 2203, 2256 |
| *Jaltomata procumbens* (Cav.) J.L. Gentry | Xaltoctoncocóyotl | Weedy | Native | Annual | Herb | Fruit | * | Seasonally | 2095, 2215 |
| *Lycianthes geminiflora* (M. Martens & Galeotti) Bitter | Hierba mora palo | Ruderal, Weedy | Native | Annual | Herb | Leaves |  | Seasonally | 2166, 2209 |
| *Physalis philadelphica* Lam. | Miltomatl | Domesticated | Native | Annual | Herb | Fruit | * | Seasonally | 2065 |
| *Solanum americanum* Mill. | Tomaquilitl | Ruderal, Weedy | Native | Annual | Herb | Leaves |  | Seasonally | 2195 |
| *Solanum glaucophyllum* Desf. | Citlaltomatl | Ruderal, Weedy | Native | Annual | Herb | Fruit |  | Seasonally | 2094 |
| *Solanum lycopersicum* L. | Jitomate | Domesticated | Native | Annual | Herb | Fruit |  | Continual | 2092 |
| *Solanum nigrescens* M. Martens & Galeotti | Tomaquilitl cimarrón | Ruderal, Weedy | Native | Annual | Herb | Leaves |  | Seasonally | 2183 |
| *Solanum tuberosum* L. | Tlahuatetl | Domesticated | Exotic | Perennial | Herb | Rizome | * | Continual | 2096 |
| *Witheringia solanacea* L'Hér. | Xaltocto | Ruderal, Weedy | Native | Annual | Herb | Leaves |  | Seasonally | 2269 |
| **Umbelliferae** |  |  |  |  |  |  |  |  |  |
| *Coriandrum sativum* L. | Cilantro | Domesticated | Exotic | Annual | Herb | Whole plant |  | Continual | 2169, 2194 |
| **Verbenaceae** |  |  |  |  |  |  |  |  |  |
| *Lippia graveolens* Kunth | Orégano | Wild | Native | Perennial | Shrub | Leaves | * | Continual | 2212, 2410 |
| **Zingiberaceae** |  |  |  |  |  |  |  |  |  |
| *Renealmia alpinia* (Rottb.) Maas | Veligmolli | Wild | Native | Perennial | Herb | Leaves, Fruit | * | Continual | 2083, 2198 |
| *Zingiber officinale* Roscoe | Gengibre | Domesticated | Exotic | Perennial | Herb | Rizome | * | Continual | 2500 |

*E. I. = Economic Importance. The species marked with this symbol (*) are sold or traded in local and regional markets.*
