# Supplementary material for: Ecological and socio-cultural factors influencing plant management in Náhuatl communities of the Tehuacán Valley, Mexico
Source: J Ethnobiol Ethnomed. 2013 Jun 2;9:39. doi: 10.1186/1746-4269-9-39 (PMC3702518; doi:10.1186/1746-4269-9-39)
Supplement: Additional file 2 — Management forms of edible plant species in Santa María Coyomeapan, México. [file 1746-4269-9-39-S2.doc]

**Appendix 2. Management forms of edible plant species in Santa María Coyomeapan, México.**

**1. *Agave salmiana* Otto ex Salm-Dyck**

Perennial, semelparous, wild and domesticated, native plant species. Its fermented sap is consumed as the beverage called ***“pulque”***. *Ex situ* management by trasplanting entire individual plants from wild populations into agricultural parcels. Also, vegetative root sprouts are taken from mother plants and planted in fields before preparing land for sowing maize. Vegetative propagules are also planted in the surounding area and rows inside parcels; available in sites close to towns. Plants are covered with ground when agricultural labors are practiced on maize. Dry leaves of agave plants are removed and burned. People said not to recognize varieties. Dedicated harvest. Temporal availability limited to the sexual reproduction period. Special tools are used for managing these agaves, particularly spatules for excaving the stem in order to make space to accumulate sap, as well as pipettes prepared with large *Lagenaria siceraria* fruits and used for sap extracting; also, machetes and special “jimador” tools are used for cutting leaves which are used for covering meat cooked in underground ovens. People recognize that some pests attack agave plants but they do not perceive that pests affect availability of this plant resource. There are no particular communitarian rules for using or accessing this plant resource. According to people, propagation of this agave species has decreased since consumption of sap and fermented sap has decreased due to its substitution by industrialized beers. However, some people still consume these products which have commercial value.

**2*. Agave obscura* Schiede**

Perennial, semelparous, native wild plant. Its flower buds consumed as greens. *In situ* management is conducted by tolerating plants in areas cleared for agriculture. Their vegetative propagules are also separated from the mother plant and established and cared in areas convenient for people. Particular care such as weeding, clearing of competing plants, and removal of dry leaves in order to increase production of flower buds are occasionally carried out. *Ex situ* management includes transplanting of vegetative propagules from the wild to agricultural parcels and homegardens. Long distances are walked for collecting propagules. Weeding, removal of dry leaves, and occasional adding of ash to the basis of the stem are practiced. No varieties are recognized. Dedicated harvest. Temporal availability limited to the sexual reproduction season. For managing and harvesting this agave people make use of machete. No pests were identified. No regulation for accessing to this plant resource was mentioned. It is interchanged in markets.

**3. *Amaranthus hybridus* L.**

Weedy annual, semelparous, native plant species. Its leaves consumed as greens (“***quilite***”). *In situ* management through tolerance inside crop fields; people use to promote its abundance by collecting and then dispersing its seeds. Weeding and removal of competing plants are practiced. Adding of inputs and irrigation benefiting maize and other crops are deliberately dedicated to indirectly benefit *A. hybridus*. *Ex situ* management is also conducted by collecting seeds at the end of an agricultural cycle, then stored in plastic bags or by hanging inflorescences in the kithchen roof to be sown in parcels during the following rainy season by throwing seeds at random. People recognize three varieties: (1) white variety (light green inflorescence and leaves), (2) purple variety with red inflorescence and leaves with purple edges, and (3) spotted, with red inflorescence and leaves with purple spots. In general, people prefer the white variety which have tender leaves and better taste. Aspect is also important, when boiled, the white variety produces green sauce (preferred), whereas the other varieties produce red sauce, which is associated with blood and not liked. However, no differential management of the varieties was observed. Sites harvested are generally close to the town. Dedicated harvest. Temporal availability limited to the early rainy season. No tools are required for management or harvesting. People recognize that this species is attaked by aphids, caterpillars and grasshoppers which cause damage of their stems and leaves; some few people said to add insecticide on this plant species when it is used for crop plants. There are no communitarian regulations for using this plant since parcels and homegardens where it is collected are mainly private property. Commercialized in markets.

**4. *Brassica campestris* L.**

Anual, weedy-ruderal, semelparous, naturalized plant species. The whole plant consumed as greens (“***quilite***”). *In situ* management through tolerance during weeding of crop fields. Its abundance is enhanced by collecting and dispersing seeds of the tolerated plants. Removal of other weeds for benefiting crop plants also benefits this species. *Ex situ* management is carried out by collecting seeds from and then stored in plastic bags. When people do not have seeds they ask permit to collect them in parcels of their relatives or neighbors. Sowing in parcels is by throwing seeds at random. Plants of this species are favored by agricultural labors invested to taking care of crop plants. People recognize two varieties, one of them called “***colesh***” with tender glabrous stems and leaves with nice flavor; the other called “***colesh teneztli***” or “***cashtelanquilitl***” (meaning “goat ***colesh***” in Náhuatl) with tomentous stems and leaves, asperous texture and bitter flavor. Plants called “***colesh***” are preferred and people collect and promote seeds of this variety, and tolerate it in parcels. Contrarily, plants called “***colesh teneztli***” are removed during weeding of agricultural fields, and their seeds never collected and sown. These plants are gathered generally in areas colse to towns. The authors observed a dedicated harvest for the “***colesh***” variety and an oportunistic harvest for the variety “***colesh teneztli***”. Temporal availability limited to the rainy season. No tools used. People recognize that caterpillars and grasshoppers cause damage to stems and leaves, but no actions are carried out to protect them. No communitarian regulations for use of this plant species was recorded. No commercialization was identified.

**5. *Canna indica* L.**

Perennial, ruderal, iteroparous, naturalized species. Its leaves are used for enveloping and flavoring food. *In situ* management by tolerance in the surrounding area of crop fields close to rivers. For increasing its density, people sow its seeds, and also use to cut old branches in order to maintain an adequated size of plants. *Ex situ* management by cultivation in homegardens, generally by transplanting complete indivudual plants and sometimes through sowing seeds. The main purpose of cultivation is to have access to this plant resource close to home since its leaves are used in preparing a high diversity of food stoves. Plants are pruned and weeding once per year and irrigated during the dry season. People recognize two varieties: (1) “***tamal panisplatl***” for prepaing the maize stove “tamales”, producing thin orange flowers, and light green long leaves, and (2) “***adorno panisplatl***”, which produces yellow flowers with larger petals and smaller leaves than the other variety and is valued as ornamental plant. Both varieties are appreciated by people, but the one with long leaves is particularly valued and used for food preparation and it is the more propagated variety. Sites of gathering are close to houses. Dedicated harvest. Available throughout the whole year. Machete and knives used for their management. No pests affecting this plant. No communitarian regulations for its use. No commercialization identified.

**6. *Cestrum nocturnum* L.**

Perennial, wild, iteroparous, native plant. Its leaves consumed as greens. *In situ* managed by tolerance in coffee plantations, benefited at least once per year by pruning, weeding, and other labors practiced on coffee plants. Plants of this species may be removed when competing with coffee plants. *Ex situ* management is conducted by transplanting complete plants or their branches from coffee plantations to homegardens. This practice occurs during the dry season and plants are irrigated until their establisment or when the rainy season delays. Plants are pruned at least once per year to promote resprouting of young leaves. Weeding is also practiced once per year. People recognize two varieties: One of them the “***buena***” (or good) produces leaves with nice flavor and abundant flowers and the other, called “***cimarrona***” produces leaves with bitter flavor and rarely blooms. People prefer the variety with good flavor but since in the last years demand for this plant has increased in markets it is more common gathering leaves of the “***cimarrona***” variety. People manage differently the two varieties, tolerating, caring and transplanting the good variety whereas removing the “***cimarrona***” variety. Gathering sites are generally close to the house. Dedicated harvest. Available the entire year. No tools used. People do not recognize any pest attaking this plant species. No communitarian regulations identified. Commercialized in markets.

**7. *Chamaedorea tepejilote* Liebm. ex Mart**

Perennial, wild, iteroparus, native plant. Consumed as greens the young male inflorescens. *In situ* management by tolerating plants in lands cleared for agriculture. Their leaves are harvested for commercialization as ornamental from January to May. Old leaves are also pruned once per year when edible young inflorescens (tepejilote) are harvested in June. Tepejilote is manually gathered with knife and for tall palms people utilize a long stick with a cord in the top. *Ex situ* management consists in trasplanting complete individual plants from forest to shaded areas of homegardens and coffee plantations. Palms cultivated in this form are smaller than palms in forest because of leaf harvesting. People also propagate palm seeds; they collect racemes with mature fruits with machete or sticks with hooks in the top. Racemes are shaken releasing their seeds in different areas of the homegarden or coffee plantations. Some people use to envelop inflorescences with mature fruits in bags and leave them dry for up 8 days in order to complete fruit maturation. Then, people spread by hand the fruits throughout selected areas. Some labors practiced in homegardens and coffe plantations (mainly weeding and irrigation) benefit tepejilote palms. People recognize four varieties: (1) “***Tepejilote metlapilli***” which produces large and thick inflorescences, (2) “***tepejilote tronquitos***” with small and thick inflorescences; (3) “***tepejilote de cafetal***” producing inflorescences with intermediate size and thickness and high production, and (4) “***tepejilote de monte***” o “***de corpus***”, with small and thin inflorescences. The first three varieties are preferred by people and have higher price in markets. The fourth variety may be occasionally used (and even commercialized) but only when the others are not available. The varieties are differentially managed. Plants of the first three varieties are transplanted from forests to homegardens and coffee plantations; however, few plants of the “***metlapilli***” variety are trasplanted because these are tall and robust plants occupying higher area than plants of the other varieties; also few plants of the “***tronquitos***” variety are trasplanted because their leaves are too small for their commercialization. The “***cafetal***” variety is the most appreciated and preferently transplanted to cultivated areas. Gathering sites are close to houses and towns, but when absent the preferred varieties people may walk long distances for collecting the “***tepejilote de monte***” variety. Neither pests nor communitarian regulations were identified. Highly commercialized.

**8. *Cleoserrata speciosa* (Raf.) H.H. Iltis**

Annual, weedy-ruderal, semelparous, native plant species. Its leaves consumed as greens (“***quilite***”). *In situ* management by tolerance in crop fields; enhanced by intentionally dispersing seeds of tolerated plants. Pods are collected, dried to the sun, and storaged in plastic bags; some plants are deliberately tolerated for ensuring seeds for the following agricultural cycle. Plants of this species are benefited by weeding and fertilization practiced on maize. *Ex situ* management through seed sowing, randomly throwing them by hand in specifically prepared areas (after removal of weeds and ground prepared) in homegardens. In corn fields seeds are also sown in specific areas (rows) particularly destined to this species. Then, in both homegardens and corn fields labors include weeding and in some cases irrigation. People do not recognize varieties. Gathering sites are close to houses. Temporal limited availability. No tools are used for harvesting seeds but for preparing land for seed sowing people utilize hoes and ploughs. Aphids and caterpillars are recognized to cause damage to leaves, but they do not consider these as important pests. No communitarian regulations were identified for using this plant species. Commercialized.

**9. *Crataegus mexicana* Moc. & Sessé ex DC.**

Perennial, iteroparous, native wild tree. Edible fruits. *In situ* management by tolerance during clearing of crop fields; young trees or resprouting plants are transplanted to the surounding areas of parcels, used as living fences. Some people practice pruning once per year. Several pests affecting this tree species are recognized, mainly beetle and butterfly larveae, but few people make use of pesticides for controlling them. *Ex situ* management is conducted by trasplanting plants from parcels to homegardens. People take into account quality of fruit produced by the “mother plant”, a well as the plant vigor. This tree is also used for grafting apple, pear, quince and peach trees; people prune branches and remove lichens growing in the bark; once per year they put lime on the basis of the tunk to protect trees against ants. No variants are recognized. Gathering sites are close to the houses and villages. Temporal availability limited to the sexual reproduction season (November to January). No tools are used for fruit harvesting but for grafting people utilize knives, machete, and brushes. Communitarian regulations prohibit cutting this tree, it requires permit by local authorities. Fruits are commercialized.

**10. *Dasylirion serratifolium* (Karw. ex Schult. f.) Zucc.**

Perennial, iteroparous, native wild plant. Its inflorescenses consumed as greens. *In situ* management through tolerance during clearance of agricultural land, benefited by weeding and fertilization practiced on crop plants. Occasionally people remove vegetative propagules growing beneath the mother plant and these propagules are placed in areas surounding the cultivated parcels. People also remove old leaves since they have the perception that such action favors vigor in inflorescens production. The bases of leaves of this plant species are also used for crafting ornamental pieces; for such purpose people select intermediate size individual plants since older plants have leaves more difficult to manipulate. People distinguish two varieties: One with light green scape with purple spots and large flower buds; the other with white scapes and smaller flower buds. The large flower buds of one variety and the good flavor of the other producing smaller flower buds are appreciated; therefore both varieties are similarly consumed and commercialized. Gathering sites are generally far from towns, requiring long distance walking. Limited temporal availability. The main tool used for gatrhering is machete. No pests were identified. Regulations for using this plant resource include federal institutions for envionmental protection (PROFEPA), as well as communitarian agreements. Both types of regulations prohibit cutting plants of this species except for some persons with permits for collecting leaves for handcrafts during specific dates for festivities. People desobeying this agreemnts may be penalized (tey have to pay $600 pesos (nearly 43 U.S. dollars). Commercialzed.

**11. *Eugenia capuli* (Schltdl. & Cham.) Hook. & Arn.**

Perennial, iteroparous, native wild tree species. It leaves, flowers and fruits consumed as spice. *In situ* management by tolerance in areas cleared for cultivating maize and coffee. Branches are pruned at least once per year to control their growing pattern in order to maintain small trees viable to be harvested. *Ex situ* management by transplanting seedlings and juvenile plants to coffee plantations, corn fields and homegardens. No varieties are distinguished. Harvesting sites are close to hosues and towns. Limited temporal availability. Machetes are used for pruning and sticks with a hook are used for harvesting branches, leaves and fruits. No pests nor communitarian rules were identified for using tis plant species but it is prohibited to cut trees. Fruits are commercialized.

**12. *Inga vera* Kunth**

Perennial, iteroparous, native wild plant. Its arile is consumed as sweet and leaves as spice. *In situ* management by tolerance in maize fields and coffee plantations where it provides shade to coffee plants. Juvenile plants and branches are collected and transplanted from one site to other inside the coffee plantations. *Ex situ* management is conducted by transplanting juvenile plants and branches from natural vegetation to coffee plantations and maize fields. Seeds from trees in plantations may also be sown. Seeds are recalcitrant and therefore can not be stored for sowing. People recognize two varieties: One of them is “***topetli de cafetal***” (“coffee plantation ***topetli***”), which has larger leaves than the other variety called “***topetli de monte***” (“wild topetli”). People prefer to propagate the “***topetli de cafetal***” variety. Gathering sites are close to town. Limited temporal availability of fruits and leaves available the whole year. Tools used for its management include machetes, knives, brushes, and shovels. People recognize that caterpillars, beetles, ants and moths affect leaves and pods of this plant species, but the only action to prevent insect attack is puting cal on stems. No communitarian agreements were identified regulating use of this plant resource. No commercialization.

**13. *Jatropha curcas* L.**

Perennial, iteroparous, naturalized plant. Their seeds are consumed. *Ex situ* management by planting branches in homegardens and coffee plantations as living fences. After cutting, branches should be planted in no more than three days, then irrigated. Juvenile plants are also transplanted among homegaredens. People occassionally sow seeds on specific areas of homegardens or in pots. No varieties are distinguished. Gathering sites are close to houses. Availability of seeds limited to the reproduction season. Toold used in its management include machetes, knives and shovels. No pests attacking this plant species are recognized. No agreements regulating use of this plant resource were identified. Commercialized.

**14. *Leucaena leucocephala* subsp. *glabrata* (Rose) Zárate**

Perennial, iteroparous, cultivated and wild plant species. Consumed as greens their young leaves and the immature seeds. *In situ* management by tolerance when land is cleared for agriculture; young trees are transplanted to the surrounding areas of the parcel. In addition, its seeds are dispersed in fallow parcels. Some caring such as putting tutor sticks for favoring straight growing of young plants is practiced. Some small fences with spiny branches are constructed around the trees in order to protect them against goats. *Ex situ* management by transplanting small trees from site to site, and taking care such as prunning and puting of cal on stems to prevent ant attacks is practiced. When dry season becomes longer, people irrigate trees of this species and eventually, people add animal dung as fertilizer. People recognize four varieties: (1) “***blanca***” (white) variety which produces light green pods and seeds of nice flavor; “***roja***” (red) variety which produces dark green leaves and red pods with stronger flavor than the “white” variety; “***prieto***” (black) variety, also called “***cimarrón***” (wild) variety, which produces dark green leaves and pods with even stronger flavor; and the “***rosa***” (pink) variety which produces sweetish seeds and pinkish pods. With the exception of the “***cimarrón***” variety, which is not consumed, the remaining varieties are consumed without clear preferences. Gathering sites of the white variety are generally close to houses and town. The “red” and “pink” varieties are trees cultivated mainly in low lands of the territory and gathering their seeds require longer distance walking. Limited temporal availability. Sticks and hooks are the only tools used for harvesting pods. People recognize that aphids and ants are main pests affecting this tree species, causing damage to young leaves and pods the only practice to protect these trees is to put cal on their stems. No rules were identified regulating use of this plant resource. Economic importance.

**15. *Litsea glauscesens* Kunth**

Perennial, iteroparous, wild, native tree species. Their leaves used as spice. *In situ* management is carried out by let standing trees when land is cleared for agriculture; also, young trees are transplanted to the surrounding areas of the parcels. Weeding and other practices for caring crop plants are deliberately practiced to benefit this species. *Ex situ* management is conducted y transplanting young plants from forests to homegardens, where people practice weeding, prunning and evantualy irrigation to benefit them. People recognize two varieties: the “smelly laurel” which produces thin, small leaves with grayish back, aromatic and small flowers. The “cimarrón laurel” variety produces wider and longer leaves with light color back, less aromatic and with larger flowers than the other variety. The smelly variety grows in pine-oak and cloud forests, whereas the “cimarrón” variety grows in humid-warm areas with tropical forest. Management is different for each variety, people prefering let standing and transplanting the “smelly” variety. Gathering sites are in general far from houses and town. Permanent temporal availability. No tools are used for gathering. No pests were recognized by people. Rules for protecting this tree species include penalization of $600 to people who cut laurel trees. Only people encharged of religious festivities are allowed to colect laurel branches, but other people have increased leave collection, given their high demand in markets, and frequently cut the tree in order to make the leaf collection easier. High economic importance.

**16. *Peperomia glabella* (Sw.) A. Dietr.**

Perennial, iteroparous, wild plant. Its leaves consumed as greens. *In situ* management through gathering leaves in wild populations. In order to maintain the plant alive, people carefully collect only part of leaves without damaging stems and roots. However, high demand of this product in the market is determining more intensive forms of gathering causing damage to plants. *Ex situ* management, one of the varieties (“tehuantequilitl” ) is cultivated. People recognize two varieties: (1) the “tequilitl” (meaning “stone quelite” in Náhuatl) variety, which produces small, thinh leaves of delicate flavor; and (2) the “tehuantequilitl” (“coyote quelite”), which produces larger and thicker leaves with stronger flavor. Although people consider the two entities as varieties, the second variety is a different species (*Peperomia maculosa*). People consider that “tequilitl” is the wild variety whereas the “tehuantequilitl” is cultivated mainly as ornamental plant. Permanent temporal availability. No tools used for gathering. No pests recognized affecting this plant species. No rules of communitarian use identified. Economic importance.

**17. *Phaseolus coccineus* L.**

Perennial, iteroparous, weedy, native plant species. Its leaves, flowers, and immature pods consumed as greens (**“*nezoquilitl”***). *In situ* management is carried out through let standing plants in crop fields, partcularly in areas surrounding the parcel. Also, people deliberately disperse its seeds in parcels where this plant species is absent. No special taking care is required but practices favoring crop plants (weeding, fertilization and ocasional prunning) also benefit to *P. coccineus*. *Ex situ* management is conducted by propagating vegetative propagules (pieces of roots) and seeds of this plant species in their homegardens mainly with ornamental purposes. No variaties were recognized by people. Gathering sites are in general long distanced from houses and town. Limited temporal availability. No tools are used for managing this plant species. Aphids and caterpillars are recognized as pests causing damage on leaves, but only ocasionally people take acctions to control them. No communitarian rules for using this plant resource were identified. Commercialized.

**18. *Phytolacca icosandra* L.**

Perennial, iteroparous, weedy-ruderal, native plant. Its leaves consumed as greens (**“molquilitl**”**)** . *In situ* management by tolerance in crop fields an may also be enhanced by dispersing its seeds, particularly in areas close to the river where people wash their clothes since fruits of this plant are saponiferous and used for this purpose. No particular caring actions on this plant species were identified. During weeding, when people have to decide which edible species should be removed, this is one of the first species cleared. No variaties are recognized by people. Gathering sites are generally close to houses and town. Limited temporal availability. No tools are required for using this plant resource. No pests were identified. No communitarian rules for using this resources wer identified. No economic importance was recorded.

**19. *Piper auritum* Kunth**

Perennial, iteroparous, wild plant. Its leaves and stems consumed as greens and spice. *In situ* management by tolerance in fallow crop fields and may also be enhanced through propagation of stems; care practices such as weeding and pruning are carried out. *Ex situ* management is carried out by propagation of stems from forest to homegardens. Satisfactory growth and increasingh availability of young leaves are achieved through pruning branches at least once per year. Weeding, adding of animal dung and irrigation are practiced during the dry season. People distinguish two varieties: (1) ***“tlanilpaquilitl casero”*** (home ***tlanilpaquilitl***) variety, which produces large light green, tender, glabrous leaves and stems, strongly aromatic; (2) “***cimarrón tlanilpaquilitl***” or wild variety, producing small leaves, stems with white glands. The second variety is really a different species (*Piper umbellatum*) which is not used. Gathering sites are close to houses and town. Permanent temporal availability. No tools are required for its use and management. Some few people recognize that caterpillars consume and affect leaves of this plant species but no actions are carried out for preventing it. No communitarian rules were identified. No commercialized.

**20. *Plantago alismatifolia* Pilg.**

Perennial, iteroparous, weedy, native plant species. Its leaves consumed as greens. *In situ* management by tolerance in crop fields and indirectly benefited from actions on crop plants (mainly weeding). *Ex situ* management ocasional people dispersing seeds of this plant species in their homegardens, mainly with the purpose to use them as dodder for hens and turkeys. People distinguish three varieties: (1) “***nenepilpitzabatl***” variety, which produces thin and long leaves; (2) “***ancho nenepilpitzabatl***” with wide leaves which is a good fodder for hens and turkeys and (3) “***cimarrón nenepilpitzabatl***” which produces redish, bitter flavored, not edible leaves. A differential management is practiced on varieties. The edible variety is tolerated and enhanced in active and fallow crop fields; the wide leaf variety is tolerated and promoted in homegardens; and the cimarrón variety is commonly removed during weeding. Gathering sites are generally close to houses and town. Limited temporal availability. No tools are required for managing this plant species. No pests were recognized affecting it. No communitarian rules were identified regulating their use. No commercialization.

**21. *Porophyllum ruderale* (Jacq.) Cass.**

Annual, semelparous, weedy-ruderal, native plant. Its leaves consumed as greens. *In situ* management by tolerance in active and fallow crop fields. People promote its abundance by dispersing its seeds in crop fields as well as practicing care actions such as weeding, tilling and fertilization. *Ex situ* managed, seeds are collected and stored for sowing them in other parcels and homegardens. Areas of some parcels are dedicated to cultivation of this plant species. Weeding, tilling, fertilization, and ocasional irrigation are practiced. People distinguish two varieties: (1) “white” variety with light green leaves and stems; and (2) “purple” variety with leaves and stems with purple areas. The white variety is cultivated and available throughout the year whereas the purple variety is tolerated and available during the dry season. Gathering sites of the white variety are close to houses and town, whereas the purple variety is available at longer distances. Management of the white variety involves tools for weeding and tilling land (machete and hoe). No pests were identified. No communitarian rules regulating use of this plant resource was identified. Commercialized.

**22. *Prunus serotina* Ehrh.**

Perennial, iteroparous, wild tree. Its fruits are consumed fresh. *In situ* managed by its tolerance during clearance of land for agriculture and in fallow parcels. Weeding, prunning and adding of clay on the stem to prevent ant attack are practiced. Young plants are transplanted to the surrounding areas of the parcels where are maintained as living fences. *Ex situ* management is practiced by transplanting young plants from forest to homegardens. In addition, seeds are deliberately dispersed in homegardens and in the surrounding area of parcels. People distinguish two varieties: (1) “***capulín casero***” (home capulín), which produces red fruits with sweet flavor and light green leaves, and the “***capulín de zorro***” (fox capulin) which produces larger black fruits with soar flavor and dark green leaves. People prefer the “home capulín” and practice differential management among varieties. The “fox capulín” is tolerated in agricultural areas since its wood is appreciated, as well as its edible seeds which are toasted and salted for consumption and have commercial value. In homegardens the “fox capulín” is generally removed, whereas the “house capulín” is tolerated, transplanted and its seeds sowed. Gathering sites are close to houses and town. Temporal availability limited to the sexual reproduction season. No tools are requied for its management. Some pests were identified determining damage on stems which produces a resin as response. Pruning and adding of cal on stems are practiced. Communitarian rules prohibit cutting capulin trees in forests. Fruits and sedes commercialized.

**23. *Quercus candicans* Née**

Perennial, iteroparous, wild tree. Its leaves are used for enveloping and spicing food. *In situ* management by let standing trees when forest is cleared for agriculture; weeding, and pruning are practiced deliberately to benefit it. *Ex situ* management, ocasionally some young plants are transplanted to homegardens and parcels close to houses. People distinguish two varieties: (1) one with large, thin, glabrous leaves with their back also glabrous, bright and light green colored; (2) the other with thicker coriaceous leaves with tomentous and darker green back than the other variety. Leaves are used for enveloping maize tamales, and for this purpose people prefer the variety with thin glabrous leaves which are more easily manipulated and because tomentous leaves confer to tamales a dark color not liked by people. No differential management of varieties was recorded. Gathering sites are generally far from houses and town. Limited temporal availability. Machete is the main tool used for managing this tree. No pests were identified. Communitarian regulations for using this plant resource include the rule of planting 10 trees and paying $600 pesos to authorities per tree cut. The rule is generally accepted. No commercialization of leaves was identified.

**24. *Raphanus raphanistrum* L.**

Annual, semelparous, weedy, naturalized plant. Its leaves consumed as greens. *In situ* managed by tolerating plants of this spefes in crop fields where it is benefited by labors such as weeding and fertilizing practiced on crop plants. *Ex situ* managed by collecting, storing and then dispersing seeds in areas used for sheep and goats raising. Also, seeds are dispersed in agricultural parcels in order to increase amount of fodder for pigs. People distinguish two variaties: (1) the “***peluda***” variety, which produces tomentous leaves with purple flowers, strong flavor and difficult digestion; and the “***lisa***” variety with smooth glabrous leaves, white flower and sweet flavor. People prefer consuming the “***lisa***” variety, but no differential management of varieties was identified. Gathering sites are generally close to houses and town. temporal availability limited to the early rainy season. No tools were identified for using this plant. Aphids and caterpillars were identified to affect this plant resource but no actions to protect it were recrded. No use rules were documented. No commercialized.

**25. *Renealmia alpinia* (Rottb.) Maas**

Perennial, semelparous, wild tree. Its leaves are used for preparing tamales as enveloping material and spice. *In situ* managed by its tolerance in coffee plantations; also, it is enhanced by extracting sprouts and transplanting them in different sites within a plantation. Weeding and pruning are practiced in order to promote vigourous resprouting. *Ex situ* management by transplanting young plants from forest to homegardens and coffee plantations. Sometimes also seeds are propagated in homegardens, where weeding, prunning and adding of ash as fertilizer are practiced. People distinguish two varieties: (1) “***velijmolli***”, which produces dark green, strongly aromatic leaves and fleshy, pinkish fruits; and (2) “***cimarrón velijmolli***” prducing light green, less aromatic leaves than the other variety. People prefer the “***velijmolli***” variety since it confers nice flavor to tamales. Gathering sites are close to houses and town. Limited temporal availability. Knives and machetes are used for its management. No pests were identified. No communitarian rules of use were recorded. Commercialized in markets.

**26. *Sambucus mexicana* C. Presl ex DC.**

Perenial iteroparous, wild tree. Its leaves are used for enveloping and spicing tamales, edible fruits. *In situ* managed tolerating trees in the surrounding areas of parcels and enhancing them by planting its branches for constructing living fences. Trees destined for using their leaves receive several prunning per year in order to maintain a short size, but only lateral branches are pruned in those trees destined to be used for wood. No variaties are distinguished. Gathering sites are close to houses and town. Permanent temporal availability. Knives and machetes are used for its management. No pests were identified. No communitarian rules were recorded. No commercialized.

**27. *Sideroxylon palmeri* (Rose) T.D. Penn.**

Perennial, iteroparous, wild tree. Fruits consumed fresh or immature as greens. *In situ* managed by its tolerance in active and fallow agricultural fields. Caring actions such as weeding, prunning and ocasional irrigation are practiced. *Ex situ* management is carried out by transplanting young plants from forest to crop fields but not to homegardens since these are large size trees. Vigor and healthy aspect of plants are taken into account for transplanting. Some plantations of ths tree species were identified, which were established because of the high demand of fruits in markets. Caring practices include weeding, prunning and ocasional irrigation. People recognize two variaties: (1) “***rounded-fruit tempesquistle***” and “***elliptic-fruit tempesquistle***”. In regional markets the “rounded-fruit tempesquistle” variety has higher demand since the “elliptic-fruit tempesquistle” variety is perceived producing higher amount of latex. Gathering sites are at intermediate distance from houses and town. Limited temporal availability. Machetes are used for management and sticks with hooks are used for gathering fruits. No pests affecting this tree species were identified. No communitarian rules of use of this plant resource were recorded. Commercialized.

**28. *Solanum americanum* Mill.**

Annual, weedy-ruderal plant species. Its leaves consumed as greens. *In situ* managed by tolerance in crop fields and indirectly benefited by practices dedicated to crop plants. *Ex situ* managed by dispersing its seeds in coffe plantations and corn fields. People recognize two varieties: (1) “***cimarrón hierbamora***” variety, which produces pubescent bitter leaves; and (2) “***real or authentic hierbamora***”, which produces glabrous stems and leaves with nice flavor. The “***real hierbamora***” variety is the one more frequently consumed and propagated as described above. Gathering sites close to houses and town. Limited temporal availability. No tools are used for managing this plant species. No pests affecting this plant species were identified by people. No communitarian rules of use of this plant resource were recorded. Economic importance.

**29. *Sonchus oleraceus* L.**

Annual, weedy-ruderal native plant species. The whole plant consumed as greens (“***quilite***”). *In situ* managed tolerated in crop fields and indirectly benefited by activities on crop plants (weeding, tilling and fertilization). Some people use to promote its abundance in fallow agricultural fields to increase its availability as fodder for goats and sheep. People recognize three varieties: (1) “white” variety, which has light green stems and wide leaves; (2) “purple” variety having stems green with purple friges; and (3) “green” or “cimarrón” variety with green, thin leaves having bitter flavor. White and purple varieties are preferred by people. Gathering sites are generally close to houses and town. Limited temporal availability. No tools used for its management. Aphids and caterpillars are the main pests recognized by people affecting this plant resource, but no action for controlling them are practiced. No communitarian rules for its use were identified. No commercialized.

**30. *Spathiphyllum cochlearispathum* (Liebm.) Engl.**

Perennial, semelparous, weedy plant. Its inflorescences consumed as greens. *In situ* managed by its tolerance in coffee plantations and benefited from activities (weeding and tilling) practiced on crop plants. Individual plants are commonly transplanted to the surrounding area of parcels. *Ex situ* managed by transplanting individual plants or vegetative parts from parcels and forest to homegardens. Weeding, tilling, removal of dry leaves and irrigation are practiced. People recognize two varieties: (1) “***elotlxóchitl***” or “***oloxóchitl***”, which produces green inflorescences with nice flavor and easily cooked; and (2) “***iztacxóchitl***”, which produces white inflorescences with bitter flavor, not consumed. People let standing and transplant the “***eloxochitl***” variety while removing the “***iztacxochitl***” variety. Gathering sites are generally close to houses and towns. Limited temporal availability. Hoe and machete are used for managing this plant. No communitarian rules were identified. Commercialized.

**31. *Tigridia pavonia* (L. f.) DC.**

Perennial, semelparous, weedy-ruderal plant, producing edible bulbs. *In situ* managed by tolerating it in crop fields and homegardens. In fallow agricultural fields people use to disperse its seeds. No varieties are recognized. Gathering sites are generally close to houses and town. Limited temporal availability. No tools are utilized for managing this plant species. No pests were identified. No rules regulating utilization of this plant resource were identified. No commercialized.

**32. *Vaccinium leucanthum* Schltdl.**

Perennial, iteroparous, wild plant, producing edible fruits consumed fresh. *In situ* managed by tolerance in surrounding areas of crop fields and inside fallow agricultural areas. Individual plants are transplanted to the surrounding area of parcels. Prunning and tilling are ocasionally practiced. No varieties are recognized by local people. Gathering sites are generally far from houses and town. Limited temporal availability. No tools are used for managing this plant species. No pests were identified. No communitarian rules were recorded. No commercialized.

**33. *Yucca elephantipes* Regel**

Perennial, iteroparous, naturalized plant. Inflorescences consumed as greens. *Ex situ* management by trasplanting vegetative parts to homegardens and surrounding áreas of parcels. Branches for propagation are left drying tos un for two weeks and then planted almost always as living fences. People use to prun branches and removing dry leaves and adding clay to stems for preventing ant attack. No varieties are recognized. Gathering sites are closet o houses. Limited temporal availability. Tools used include hooks and machetes. Some beetles are recognized to cause damage to inflorescences but no actions are practiced to prevent it. No rules for using this species was identified. Its inflorescences are commercialized in the regional market of Ajalpan.
